# Effective Biobased Phosphorus Flame Retardants from Starch-Derived *bis*-2,5-(Hydroxymethyl)Furan

**DOI:** 10.3390/molecules25030592

**Published:** 2020-01-29

**Authors:** Bob A. Howell, Xiaorui Han

**Affiliations:** Department of Chemistry and Biochemistry, Center for Applications in Polymer Science, Central Michigan University, Mt. Pleasant, MI 48858-0001, USA; han1x@cmich.edu

**Keywords:** biobased flame retardants, Phospha-Michael addition, furan additives, phosphorus derivatives of bisphenol and tetrabromobisphenol acrylates, green polymer additives, nontoxic flame retardants

## Abstract

A series of biobased phosphorus flame retardants has been prepared by converting starch-derived *bis*-2,5-(hydroxymethyl)furan to the corresponding diacrylate followed by Michael addition of phosphite to generate derivatives with phosphorus moieties attached via P–C bonds. All compounds behave as effective flame retardants in DGEBA epoxy resin. The most effective is the DOPO derivative, 2,5-di[(3-dopyl-propanoyl)methyl]furan. When incorporated into a DGEBA blend at a level to provide 2% phosphorus, a material displaying a LOI of 30, an UL 94 rating of V0 and a 40% reduction in combustion peak heat release rate compared to that for resin containing no additive is obtained. The analogous compounds generated from bisphenol A and tetrabromobisphenol A exhibit similar flame-retarding properties.

## 1. Introduction

Polymeric materials have made a huge contribution to the development of modern society [1,2]. Apart from naturally-occurring polymers and modifications, these have generally been derived from petrochemicals. More recently, the generation of biobased polymers has gained interest [3]. For most applications polymeric materials must be flame retarded. Despite the popularity and effectiveness of traditional organohalogen flame retardants, in particular brominated aromatics, there has been a strong move away from the use of these materials. These compounds may be converted to volatile and toxic dioxins at high temperature [4]. However, the much larger concern arises from the migration of these compounds from a polymer matrix into which they have been incorporated. This is particularly a problem for waste items discarded in a landfill [5]. The flame retardants escape into the environment, are not biodegradable, bioaccumulate and pose risks to human health. Widespread human exposure to these materials has occurred and has been associated with a variety of disease states, most arising from endocrine disruption [6,7,8,9]. The negative impacts of human exposure to these compounds has promoted public pressure for the restriction of their use. This has occurred by both regulation and voluntary removal from the market [10,11,12]. The best candidates to replace organohalogen flame retardants are organophosphorus compounds [13,14]. In general, the toxicity of organophosphorus compounds is much lower than that observed for organohalogen counterparts [15,16,17]. Further, the effectiveness of organophosphorus flame retardants may be enhanced by the presence of compounds containing other elements, most notably sulfur [18,19,20,21], nitrogen [22,23,24,25,26,27], boron [28,29], or silicon [30,31,32]. Often the effectiveness of the presence of two flame-retarding compounds is touted as being synergistic. However, this is almost never the case. Usually, the impact of the two compounds on the suppression of flammability is not even additive. 

The development of polymer additives from naturally-occurring renewable materials is increasingly important [33]. The precursors to these materials are readily available, renewable annually, environmentally-benign, usually biodegradable, and generally nontoxic. They may be obtained from a variety of sources [34]. Further, the utilization of these materials is independent of the cost or availability of petrochemicals. Most prominently, the development of new flame retardants from biosources has been a focus [35,36,37,38,39,40,41,42,43,44,45]. Plant sources have long been the origin of many commercial materials [46]. More recently there has been an emphasis on starch from seed grains. A major source of starch, particularly in the United States, has been corn [47,48]. Starch may be hydrolyzed to afford glucose which can converted to many useful materials mainly through 5-hydroxymethylfurfural (HMF) [49,50,51,52]. Glucose may be reduced and then dehydrated to provide isosorbide, a diether diol, suitable for the generation of a range of phosphorus flame retardants [38,39,40]. Glucose, and other carbohydrates, may also serve as a source of *bis*-2,5-(hydroxymethyl)furan (BHMF). This difunctional alcohol has been used for the production of poly(ester)s [53,54,55,56] and plasticizers [57]. In this case, it was converted to the corresponding diacrylate. Michael addition of phosphite to the acrylate was then used to generate a series of bioderived phosphorus flame retardants containing P–C bonds [58,59,60].

## 2. Results and Discussion

**Scheme 1:**
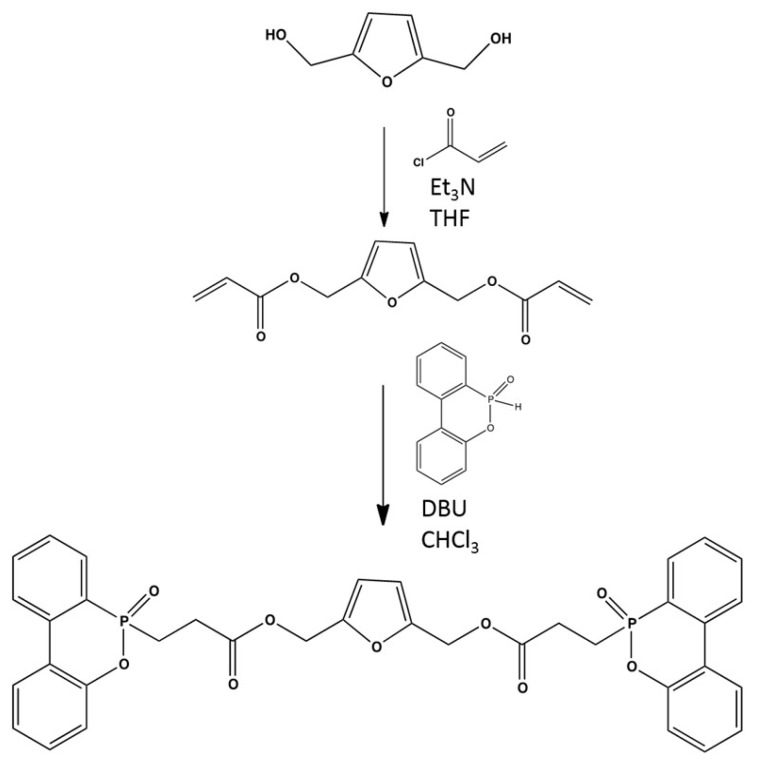
Synthesis of 2,5-Di[(3-dopylpropanoyl)methyl]furan (DDMF).

The development of effective flame retardants from renewable biosources offers several advantages over traditional processes based on petrochemicals and is receiving increasing attention. The impetus for this development is a heightened concern for environmental quality and an increased awareness of the toxicity of traditional flame retardants. In this case, bioderived *bis*-2,5-(hydroxymethyl)furan has been utilized as the base for the generation of a series of phosphorus flame retardants. The diol was first converted to the diacrylate. Michael addition of phosphite to the diacrylate afforded the corresponding phosphorus derivatives [58]. This is illustrated in Scheme 1 for the preparation the DOPO derivative (DDMF). 

The corresponding diethyl-(DEMF) and diphenylphosphite (DPMF) adducts were prepared in an analogous manner. The structures for all compounds were rigorously established using spectroscopic and thermal methods (see Experimental section); spectra may be found in Supplemental Material). Pertinent infrared absorptions for these compounds may be found in Table 1, proton NMR chemical shifts in Table 2 and the corresponding carbon-13 chemical shifts in Table 3. 

**Figure 1:**
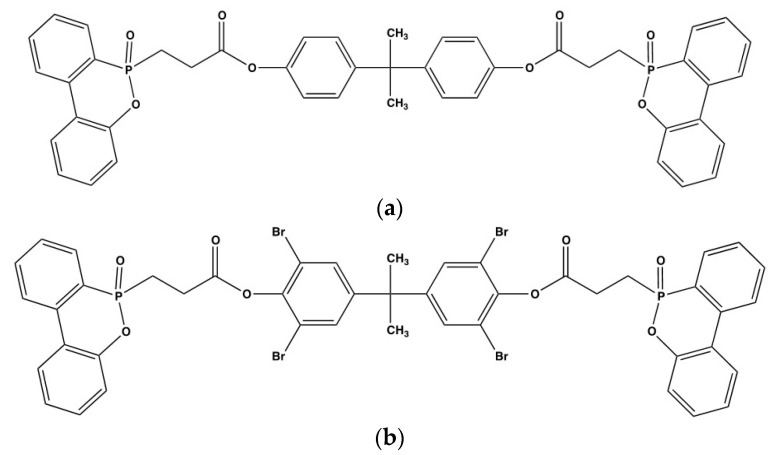
Structures for the DOPO adducts of bisphenol A and Tetrabromobisphenol A diacrylates. (**a**) 2,2-Di[4-(3-dopylpropanoyl)phenyl]propane (DDPP); (**b**) 2,2-Di[3,5-dibromo-4-(3-dopylpropanoyl)phenyl]propane (DBDPP).

For comparison the corresponding DOPO derivatives of bisphenol A and tetrabromobisphenol A were prepared. Structures are shown in Figure 1.

For assessment of the effectiveness of these compounds, blends with DGEBA epoxy at levels to provide 1% or 2% phosphorus and in two cases 5% phosphorus were prepared [58]. The flammability of the blends was determined using limiting oxygen index (LOI), microscale combustion calorimetry (MCC), and the underwriter Laboratories vertical burn test (UL94). Results are presented in Table 4.

As may be noted the presence of any of the additives sharply decreases the flammability compared to that for epoxy containing no flame retardant (LOI 19, PHRR 498 W/g, unrated in UL 94). For the derivatives of *bis*-2,5-(hydroxymethyl)furan, the DOPO adduct is the most effective. At a loading to provide 2% phosphorus in the blend, an LOI of 29.1, a PHRR of 305 W/g and a UL 94 rating of V0 are observed. Derivatives of DOPO (low level of oxygenation at phosphorus) are predominately gas-phase active and function by extruding PO radical to the gas phase to quench combustion propagating reactions [61,62,63,64,65,66]. On the other hand, derivatives of diethyl- and diphenylphosphite are probably not gas-phase active and a higher loading (5% phosphorus) of these additives is required to achieve comparable flame retardancy. 

A comparison of the effectiveness of the DOPO derivatives of the diacrylates of *bis*-2,5-(hydroxymethyl)furan, bisphenol A and tetrabromobisphenol A is presented in Table 5. 

At a loading to provide 2 wgt% phosphorus, the impact of the additives is quite similar: LOI of 30, 40% reduction in PHRR and a V0 UL 94 rating. Certainly, the impact of the furan derivative is comparable to that of the corresponding bisphenyl compounds. It is interesting that the brominated bisphenyl compound is little more effective than the analogous compound containing no bromine [67,68]. This may suggest that phosphorus is primarily responsible for the flame retardant effect.

## 3. Materials and Methods

### 3.1. Materials

Common solvents and reagents were from commercial sources and were purified as appropriate prior to use [58]. *bis*-2,5-(Hydroxymethyl)furan (BHMF) was supplied by PennAkem, LLC (Nashville, TN, USA). Diphenylchlorophossphate was provided by ICL-IPAmerica, Inc. (Ardsely, NY, USA) 9,10-Dihydro-9-oxaphosphaphenanthrene-10-oxide (DOPO) was from TCI (Portland, OR USA). The diglycidyl ether of bisphenol A (DGEBA) was supplied by the Dow Chemical Company (Midland, MI, USA). 

### 3.2. Methods and Instrumentation

Instrumentation and methods for characterization using spectroscopic, chromatographic and thermal techniques have been described previously [58]. Infrared spectra were obtained by attenuated total reflectance (ATR) using a Thermo Scientific (Waltham, MA, USA) Nicolet 380 FT-IR spectrophotomerter. Absorptions were recorded in wavenumbers (cm^−1^), and absorption intensities were classified in the usual fashion as very weak (vw), weak (w), medium (m), strong (s), and very strong (vs) relative to the strongest band in the spectrum. Nuclear magnetic resonance (NMR) spectra were obtained using a 5–15% solution in deuterochloroform or dimethylsulfoxide-*d*_6_ and a Varian (Santa Clara, CA, USA) Mercury 300 MHz or an INOVA 500 MHz spectrometer. Proton and carbon chemical shifts are reported in parts per million (*δ*) with respect to tetramethylsilane (TMS) as an internal reference (*δ* = 0.00). Phosphorus chemical shifts are in *δ* with respect of triphenylphosphate as internal reference (δ = −18.00). Electrospray ionization mass spectrometry (ESI-MS) was carried out using a Waters (Milford, MA, USA) Acquity/LCT Premier XE unit with samples introduced as dilute solutions in acetonitrile/water. Matrix assisted laser desorption ionization (MALDI) time of flight mass spectrometry was performed using a Bruker (Billerica, MA, USA) Daltonics Autoflex unit and 2,6-dihydroxybenzoic acid as matrix. Thermal transitions were determined by differential scanning calorimetry (DSC) using a TA instruments (New Castle, DE, USA) Q2000 instrument. Samples, contained in standard aluminum pans, were analyzed at a heating rate of 5 or 10 °C min^−1^. The cell was subject to a constant purge of dry nitrogen at 50 cm^3^ min^−1^. Thermogravimetry was performed using a TA instruments Q500 instrument. Typically, a heating rate of 5 or 10 °C min^−1^ was used. Samples (4–10 mg) were contained in a platinum pan. The sample compartment was purged with dry nitrogen at 50 cm^3^ min^−1^ during analysis. Peak heat release rates were determined using a Fire Testing Technology, Ltd. (East Grinstead, UK) (FTT) microscale combustion calorimeter, model FAA-PCFC. Limiting oxygen index values were determined using an FTT Oxygen Index unit. Vertical burn tests were conducted in an FTT UL 94 test chamber.

### 3.3. Test Specimen

Standard plaques for flammability testing were prepared from DGEBA epoxy using 2-ethyl-4-methylimidazole as hardener [58]. Samples were prepared by dissolving sufficient additive to provide a loading of one percent phosphorus in digycidyl ether of bisphenol A (DGEBA) epoxy at 90 °C. Hardener 2-ethyl-4-methylimidazole was added, mixed thoroughly, and the whole was poured into Teflon molds of appropriate dimensions which had been allowed to equilibrate at 95 °C. The blends were cured initially at 95 °C for 1.5 h and then at 130 °C for 1.5 h. The samples were then allowed to cool slowly (0.6 °C/min) to room temperature.

### 3.4. Flammability Testing 

Flammability testing was accomplished using standard methods: limiting oxygen index (LOI; ASTM D2863-13), microscale combustion and calorimetry (MCC; ASTM D7309-07a), and the UL 94 vertical burn test (ASTM D3801-06). 

### 3.5. Synthesis

The *bis-*acrylates of *bis*-2,5-(hydroxymethyl)furan (BHMF), bisphenol A (BPA), and tetrabromobisphenol A (TBBPA) were prepared by treating the appropriate diol with acryloyl chloride in the presence of triethylamine [58].

*bis-2,5-(Hydroxymethyl)furan Diacrylate.* To a stirred solution of 21.60 g (0.17 mol) of *bis*-2,5-(hydroxymethyl)furan and 50 mL (0.37 mol) of triethylamine, 200 mL of anhydrous THF maintained near 0 °C (external ice bath) was added, dropwise, over a period of 2 h, a solution of 30 mL (0.37 mol) of acryloyl chloride in 150 mL of anhydrous THF. The resulting mixture was allowed to warm to room temperature and stirred for 6 h. Water (200 mL) was added dropwise, followed by 200 mL diethyl ether. The layers were separated and the ether solution was washed, successively, with three 80-mL portions of 5% aqueous hydrochloric acid solution, 80 mL of 5% aqueous sodium hydroxide solution and 80 mL of saturated aqueous sodium chloride solution. The ether solution was dried over anhydrous sodium sulfate and the solvent was removed by rotary evaporation at reduced pressure. The crude product was purified by column chromatography (silica gel; 3:1 ethyl-acetate/hexane as eluent) to provide 29.08 g (72.4% yield) of the diacrylate as a colorless oil: T_dec_ 128 °C (TGA); FTIR (cm^−1^, ATR) 3045 (w) C_sp2_–H, 2956 (w), 2834 (w) C_sp3_–H, 1721 (vs) ester carbonyl, 1634 (w) C=C, 1613 (m) aromatic nucleus, 1182 (vs) C-O; ^1^H-NMR (δ, DMSO-*d_6_*) 5.02 (s, methylene protons), 5.78–6.39 (m, 6H, ABX pattern over a singlet, furan and phenyl protons); ^13^C-NMR (δ, DMSO-*d_6_*) 58.3 (methylene carbon atoms), 112.5, 150.5 (furan carbon atoms), 128.3, 132.8 (vinylic carbon atoms), 165.6 (carbonyl carbon atoms); MS (ESI, *m/z*) 259.03 [M + Na]^+^, 275.02 [M + K]^+^. 

Other acrylates were prepared in an analogous manner.

*Bisphenol A Diacrylate.* M.p. 89 °C (DSC); T_dec_ 431 °C (TGA); FTIR (cm^−1^, ATR) 3086 (w) C_sp2_–H, 2971 (m) 2873 (w) C_sp3_–H, 1750 (s) ester carbonyl, 1633(w) C=C, 1508 (s) aromatic nucleus; ^1^H-NMR (δ, CDCl_3_) 1.69 (s, 6H, methyl protons), 5.96–6.66 (ABX pattern, 6H, vinylic protons), 7.16 (AB pattern, 8H, aromatic protons); ^13^C-NMR (δ, CDCl_3_) 31.0 (methyl carbon atoms), 42.5 (quaternary carbon atoms), 128.0, 132.5 (vinylic carbon atoms), 120.9, 127.9, 147.9, 148.5 (aromatic carbon atoms), 164.6 (carbonyl carbon atoms).

*Tetrabromobisphenol A Diacrylate*. T_dec_ 345 °C (TGA); FTIR (cm^−1^, ATR) 3098 (w) C_sp2_–H, 2971 (w), 2873 (w) C_sp3_–H, 1750 (s) ester carbonyl, 1633 (w) C=C, 1605 (m) aromatic nucleus, 1182 (s) C–O; ^1^H-NMR (δ, CDCl_3_) 1.62 (s, methyl protons), 6.09–6.78 (ABX pattern, 6H, vinylic protons), 7.40 (s, 4H, aromatic protons); ^13^C-NMR (δ, CDCl_3_) 29.8 (methyl carbon atoms), 43.1 (quaternary carbon atoms), 126.2, 136.1 (vinylic carbon atoms), 117.8, 130.7, 144.3, 150.4 (aromatic carbon atoms), 162.3 (carbonyl carbon atoms).

The acrylate esters of *bis*-2,5-(hydroxymethyl)furan, bisphenol A and tetrabromobisphenol A were converted to phosphorus derivatives by Michael addition of phosphites using a previously described procedure [58].

*2,5-Di[(3-dopylpropanoyl)methyl]furan (DDMF).* A solution of 20.61 g (0.095 mol) of DOPO, 5.7 mL (0.038 mol) of 1,8-diazabicycloundec-7-ene and 9.01 g (0.038 mol) of *bis*-2,5-(hydroxymethyl)furan diacrylate in 75 mL of chloroform was stirred at 65 °C for 4 h. The solution was allowed to cool to room temperature and washed, successively with three 20-mL portions of 5% aqueous hydrochloric acid solution, 20 mL of 5% aqueous sodium hydroxide solution and 20 mL of saturated aqueous sodium chloride solution. The chloroform solution was dried over anhydrous sodium sulfate and the solvent was removed by rotary evaporation at reduced pressure. The crude product was purified by column chromatography (silica gel; 5:3 ethyl acetate/hexane as eluent) to afford 23.45 g (92.10% yield) of DDMF as a white solid: m.p. 47 °C (DSC), T_dec_ 352 °C (TGA); FTIR (cm^−1^, ATR) 3071 (w) C_sp2_–H, 2923 (w) C_sp3_–H, 1732 (vs) ester carbonyl, 1598 (w) aromatic nucleus, 1235 (vs) C–O, 1197 (vs) P=O, 910 (s), 757 (s) P–O–C; ^1^H-NMR (δ, CDCl_3_) 2.36 (m, 4H, methylene protons adjacent to phosphorus), 2.68 (m, 4H, methylene protons adjacent to carbonyl groups), 4.98 (s, 4H, methylene protons adjacent to oxygen and a furan nucleus), 6.31 (s, 2H, furan aromatic protons), 7.18–7.98 (m, 16H, dopyl protons); ^13^C-NMR (δ, CDCl_3_) 23.2, 26.3, 58.1 (methyl carbon atoms), 111.9, 120.4, 123.0, 123.9, 124.8, 125.2, 128.6, 130.1, 130.7, 133.5, 135.8, 148.5, 149.2, 164.0 (furan and dopyl carbon atoms), 171.8 (carbonyl carbon atoms); MS (ESI, *m/z*), 669.13 [M + H]^+^, 691.11 [M + Na]^+^, 707.08 [M + K]^+^.

Other adducts were prepared in an analogous manner.

*2,5-Di[(3-diethylphosphonatopropanoyl)methyl]furan (DEMF).* T_dec_ 181 °C (TGA); FTIR (cm^−1^, ATR) 3066 (w) C_sp2_–H, 2953 (w) and 2926 (w) C_sp3_–H, 1737 (vs) ester carbonyl, 1596 (w) aromatic nucleus, 1232 (vs) C–O, 1208 (vs) P=O, 907 (s), 754 (s) P–O–C; ^1^H-NMR (δ, CDCl_3_) 1.26 (m, 12H, methyl protons), 2.02 (m, 4H, methylene protons adjacent to phosphorus), 2.58 (m, 4H, methylene protons adjacent to carbonyl groups), 4.03 (m, 8H, methylene protons on ethyl groups), 4.99 (s, 4H, methylene protons adjacent to oxygen and a furan nucleus), 6.32 (s, 2H, furan protons); ^13^C-NMR (δ, CDCl_3_) 16.4 (methyl carbon atoms), 20.9 (methylene carbon atoms adjacent to phosphorus), 27.4, 58.3, 62.0 (methylene carbon atoms), 111.7, 149.9 (furan carbon atoms), 171.6 (carbonyl carbon atoms); MS (ESI, *m/z*), 513.13 [M + H]^+^, 535.07 [M + Na]^+^, 551.09 [M + K]^+^.

*2,5-Di[(3-diphenylphosphonatopropanoyl)methyl]furan (DPMF).* T_dec_ 198 °C (TGA); FTIR (cm^−1^, ATR) 3065 (w) C_sp2_–H, 2930 (w) C_sp3_–H, 1740 (vs) ester carbonyl, 1598 (w) aromatic nucleus, 1186 (vs) C–O, 1162 (vs) P=O, 927 (s), 762 (s) P–O–C; ^1^H-NMR (δ, CDCl_3_) 2.46 (m, 4H, methylene protons adjacent to phosphorus), 2.85 (m, 4H, methylene protons adjacent to carbonyl groups), 5.02 (s, 4H, methylene protons adjacent to oxygen and a furan nucleus), 6.37 (s, 2H, furan protons), 7.14 and 7.29 (m, 20H, phenyl protons); ^13^C-NMR (δ, CDCl_3_) 21.4 (carbon atoms adjacent to phosphorus), 27.4 (carbon atoms adjacent to carbonyl groups), 58.6 (carbon atoms adjacent to oxygen), 111.9, 149.9 (furan carbon atoms), 120.5, 125.3, 129.8, 150.1 (phenyl carbon atoms), 171.2 (carbonyl carbon atoms); MS (ESI, *m/z*) 705.11 [M + H]^+^, 727.07 [M + Na]^+^, 743.05 [M + K]^+^.

*2,2-Di[4-(3-dopylpropanoyl)phenyl]propane (DDPP).* Mp 79 °C (DSC), T_dec_ 373 °C (TGA); FTIR (cm^−1^, ATR) 3052 (w) C_sp2_–H, 2973 (m), 2862 (w) C _sp3_–H, 1750 (s) ester carbonyl, 1625 (w) aromatic nucleus, 1187 (s) P=O, 1173 (s) C–O, 901 (m), 743 (m) P–O–C; ^1^H-NMR (δ, CDCl_3_) 1.61 (s, 6H, methyl protons), 2.59 (m, 4H, methylene protons adjacent to phosphorus), 2.90 (m, 4H, methylene protons adjacent to carbonyl groups), 6.91–7.17 (m, 8H, bisphenol protons), 7.21–8.01 (m, 16H, dopyl protons); ^13^C-NMR (δ, CDCl_3_) 23.5 (carbon atoms adjacent to phosphorus), 24.3 (carbon atoms adjacent to carbonyl groups), 30.9 (methyl carbon atoms), 42.5 (quaternary carbon atoms), 120.5–128.6, 130.1, 130.8, 133.6, 135.8, 148.0, 148.4, 148.9 (aromatic carbon atoms), 170.4 (carbonyl carbon atoms); ^31^P-NMR (δ, CDCl_3_) 36.0; MS (ESI, *m/z*) 769.10 [M + H]^+^, 791.15 [M + Na]^+^, 807.11 [M + K]^+^.

*2,2-Di[3,5-dibromo-4-(3-dopylpropanoyl)phenyl]propane (DBDPP).* M.p. 108 °C (DSC); T_dec_ 321 °C (TGA); FTIR (cm^−1^, ATR) 3064 (w) C_sp2_–H, 2970 (w), 2919 (w) C _sp3_–H, 1770 (vs) ester carbonyl, 1587 (w) aromatic nucleus, 1249 (vs) C–O, 1123 (vs) P=O, 917 (s), 765 (s) P–O–C; ^1^H-NMR (δ, CDCl_3_), 1.58 (s, 6H, methyl protons), 2.53 (m, 4H, methylene protons adjacent to phosphorus), 3.08 (m, 4H, methylene protons adjacent to carbonyl groups), 7.32 (s, 4H, protons of dibromophenyl groups), 7.21–7.98 (m, 16H, dopyl protons); ^13^C-NMR (δ , CDCl_3_) 24.1 (carbon atoms adjacent to phosphorus), 26.3 (carbon atoms adjacent to carbonyl groups), 30.6 (methyl carbon atoms), 42.4 (quaternary carbon atoms), 117.4 (bromoaryl carbon atoms), 120.3, 121.9, 123.2, 124.1, 124.8, 125.3, 128.2, 128.8, 129.0, 130.1, 130.8, 133.6, 135.8, 144.3, 149.6 (aromatic carbon atoms), 168.2 (carbonyl carbon atoms); ^31^P-NMR (δ, CDCl_3_) 36.0; MS (ESI, *m/z*) 1084.40 [M + H]^+^, 1106.78 [M + Na]^+^, 1122.75 [M + K]^+^.

## 4. Conclusions

Bioderived *bis*-2,5-(hydroxymethyl)furan has been utilized as a base for the generation of a series of phosphorus compounds. The dihydroxy furan was converted to the corresponding *bis*-acrylate which was, in turn, subjected to Michael addition of phosphite to generate phosphorus derivatives with phosphorus linked through a P–C bond. These compounds act as effective flame retardants in DGEBA epoxy. The DOPO derivative, 2,5-di[(3-dopylpropanoyl)methyl]furan, is the most effective. At a loading to provide 2% phosphorus in a DGEBA blend an LOI of 30, a UL94 rating of V0 and a 40% reduction in peak heat release rate for combustion compared to that for resin containing no additive is observed. This performance is comparable to that observed for the analogous derivatives of bisphenol A and tetrabromobisphenol A diacrylates.

## Figures and Tables

**Table 1:** Characteristic Infrared Absorptions (cm^−1^) for Phosphorus Derivatives of 2,5-*bis*(Hydroxymethyl)furan Diacrylate.

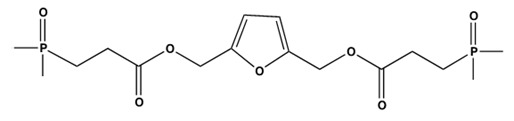							
Phosphorus Substituent	C_sp3_–H	C_sp2_–H	C=O	Aromatic Nucleus	C–O–C	P=O	P–O–C
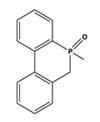	3071	2923	1732	1598	1235	1197	910, 757
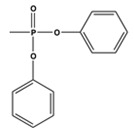	3065	2930	1740	1598	1186	1162	927, 762
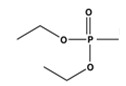	3066	2926, 2953	1737	1596	1232	1208	907, 754

**Table 2:** Characteristic ^1^H-NMR chemical shifts for phosphorus derivatives of 2,5-*bis*(Hydroxymethyl)furan diacrylate. ^a^

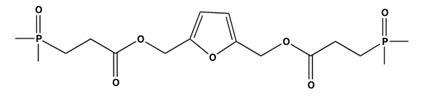				
Phosphorus Substituent	Methylene Protons Adjacent to Phosphorus	Methylene Protons Adjacent to Carbonyl	Methylene Protons Adjacent to Furan Nucleus	Furan Protons
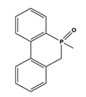	2.36	2.68	4.98	6.31
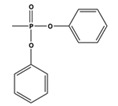	2.46	2.85	5.02	6.37
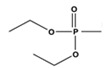	2.02	2.58	4.99	6.32

^a^ In δ units with respect the resonance of tetramethylsilane as internal reference.

**Table 3:** Characteristic ^13^C-NMR chemical shifts for phosphorus derivatives of 2,5-*bis*(Hydroxymethyl)furan diacrylate. ^a^

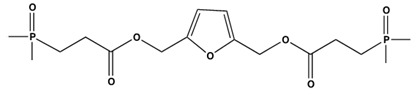					
Phosphorus Substitutent	Carbon Atoms Adjacent to Phosphorus	Carbon Atoms Adjacent to Carbonyl	Carbon Atoms Adjacent to Oxygen and Furan Nucleus	Furan Carbon Atoms	Ester Carbonyl Carbon Atoms
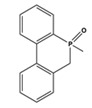	23.2	26.3	58.1	111.9, 164.0	171.8
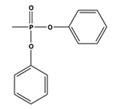	21.4	27.4	58.6	111.9, 149.9	171.2
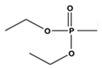	20.9	27.4	58.3	111.7, 149.9	171.6

^a^ In δ units respect the resonance of tetramethylsilane as internal reference.

**Table 4:** Flammability data for blends of flame retardants with DGEBA epoxy resin.

Flame Retardants Structure	Loading of Additive (wgt%) ^a^	Loading of Phosphorus (wgt%) ^b^	LOI ^c^	UL-94 Designation	PHRR (W/g) ^d^
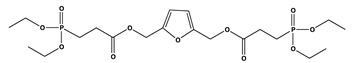	8	1	24.2	-	391
	17	2	24.6	-	363
	41	5	27.0	V1	350
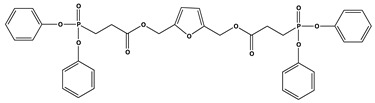	11	1	24.5	-	420
	23	2	25.1	-	333
	57	5	26.9	V0	246
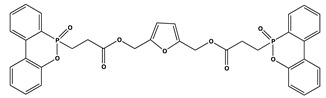	11	1	28.4	V1	344
	22	2	29.1	V0	305
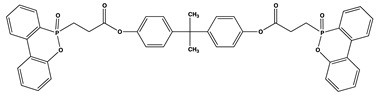	12	1	28.6	V0	449
	25	2	30.3	V0	330
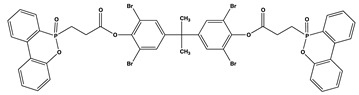	17	1	28.1	V0	320
	35	2	30.4	V0	262

^a^ Weight percent of additive in DGEBA blend. ^b^ Weight percent of phosphorus in DGEBA blend. ^c^ Limiting Oxygen Index (LOI). ^d^ Microscale Combustion Calorimetry.

**Table 5:** Flammability of blends (2% Phosphorus) of the 9,10-Dihydro-9-oxaphosphaphenanthrene-10-oxide (DOPO) adducts of 2,5-*bis*(Hydroxymethyl)furan diacrylate, bisphenol A Diacrylate, and tetrabromobisphenol A diacrylate with DGEBA epoxy.

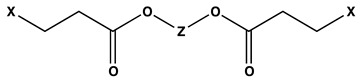				
Z	X	LOI	UL-94 Designation	PHRR (W/g)
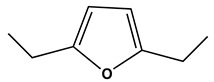	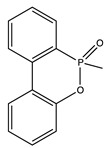	29.1	V0	305
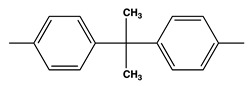		30.3	V0	330
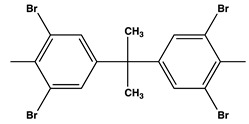		30.4	V0	262

## Supplementary Materials

The following are available online: Figure S1: The ^1^H-NMR Spectrum for Bisphenol A Diacrylate (BPADA), Figure S2: The ^13^C-NMR spectrum for bisphenol A diacrylate (BPADA), Figure S3: Infrared spectrum of bisphenol A diacrylate (BPADA), Figure S4: The ^1^H-NMR spectrum of 2,2-di[4-(3-dopylpropanoyl)phenyl]propane (DDPP), Figure S5: The ^13^C-NMR spectrum of 2,2-di[4-(3-dopylpropanoyl)phenyl]propane (DDPP), Figure S6: The DEPT NMR spectrum of 2,2-di[4-(3-dopylpropanoyl)phenyl]propane (DDPP), Figure S7: The gCOSY NMR spectrum of 2,2-di[4-(3-dopylpropanoyl)phenyl]propane (DDPP), Figure S8: The ^31^P-NMR spectrum of 2,2-di[4-(3-dopylpropanoyl)phenyl]propane (DDPP), Figure S9: ESI mass spectrum of 2,2-di[4-(3-dopylpropanoyl)phenyl]propane (DDPP), Figure S10: The ^1^H-NMR spectrum for tetrabromobisphenol A diacrylate (BBPADA), Figure S11: The ^13^C-NMR Spectrum for Tetrabromobisphenol A Diacrylate (BBPADA), Figure S12: The Infrared Spectrum of of tetrabromobisphenol A diacrylate (BBPADA), Figure S13: The ^1^H-NMR spectrum 2,2-di[3,5-dibromo-4-(3-dopylpropanoyl)phenyl]propane (DBDPP), Figure S14: The gCOSY NMR spectrum for 2,2-di[3,5-dibromo-4-(3-dopylpropanoyl)phenyl]propane (DBDPP), Figure S15: The DEPT NMR spectrum for 2,2-di[3,5-dibromo-4-(3-dopylpropanoyl)phenyl]propane (DBDPP), Figure S16: The ^13^C-NMR spectrum for 2,2-di[3,5-dibromo-4-(3-dopylpropanoyl)phenyl]propane (DBDPP), Figure S17: The HSQCAD NMR spectrum for 2,2-di[3,5-dibromo-4-(3-dopylpropanoyl)phenyl]propane (DBDPP), Figure S18: The infrared spectrum of 2,2-di[3,5-dibromo-4-(3-dopylpropanoyl)phenyl]propane (DBDPP), Figure S19: The ESI mass spectrum of 2,2-di[3,5-dibromo-4-(3-dopylpropanoyl)phenyl]propane (DBDPP), Figure S20: The ^1^H-NMR spectrum for 2,5-*bis*-(hydroxymethyl)furan diacrylate (BHFDA), Figure S21: The ^13^C-NMR spectrum for 2,5-*bis*-(hydroxymethyl)furan diacrylate (BHFDA), Figure S22: The infrared spectrum for 2,5-*bis*-(hydroxymethyl)furan diacrylate (BHFDA), Figure S23: Mass spectrum for 2,5-*bis*-(hydroxymethyl)furan diacrylate (BHFDA), Figure S24: The ^1^H-NMR spectrum for 2,5-di[(3-dopylpropanoyl)methyl]furan (DDMF), Figure S25: The gCOSY NMR spectrum for 2,5-di[(3-dopylpropanoyl)methyl]furan (DDMF), Figure S26: The ^13^C-NMR spectrum for 2,5-di[(3-dopylpropanoyl)methyl]furan (DDMF), Figure S27: The DEPT NMR spectrum for 2,5-di[(3-dopylpropanoyl)methyl]furan (DDMF), Figure S28: The HSQCDA NMR spectrum for 2,5-di[(3-dopylpropanoyl)methyl]furan (DDMF), Figure S29: The infrared spectrum of 2,5-di[(3-dopylpropanoyl)methyl]furan (DDMF), Figure S30: The ESI mass spectrum of 2,5-di[(3-dopylpropanoyl)methyl]furan (DDMF), Figure S31: The ^1^H-NMR spectrum for di[(3-diethylphosphonatopropanoyl)methyl]furan (DEMF), Figure S32: The ^13^C-NMR spectrum for di[(3-diethylphosphonatopropanoyl)methyl]furan (DEMF), Figure S33: The infrared spectrum of di[(3-diethylphosphonatopropanoyl)methyl]furan (DEMF), Figure S34: The ESI mass spectrum of di[(3-diethylphosphonatopropanoyl)methyl]furan (DEMF), Figure S35: The ^1^H-NMR spectrum for di[(3-diphenylphosphonatopropanoyl)methyl]furan (DPMF), Figure S36: The ^13^C-NMR spectrum for di[(3-diphenylphosphonatopropanoyl)methyl]furan (DPMF), Figure S37: The infrared spectrum for di[(3-diphenylphosphonatopropanoyl)methyl]furan (DPMF), Figure S38: The ESI mass spectrum for di[(3-diphenylphosphonatopropanoyl)methyl]furan (DPMF).
