# Supplementary material for: Effective Biobased Phosphorus Flame Retardants from Starch-Derived bis-2,5-(Hydroxymethyl)Furan
Source: Molecules. 2020 Jan 29;25(3):592. doi: 10.3390/molecules25030592 (PMC7037623; doi:10.3390/molecules25030592)
Supplement: Supplementary file 1 [file molecules-25-00592-s001.pdf]

Effective Biobased Phosphorus Flame Retardants from Starch-derived *bis*-2,5-(Hydroxymethyl)furan

Bob A. Howell and Xiaorui Han
Center for Applications in Polymer Science
Department of Chemistry and Biochemistry
Central Michigan University
Mt. Pleasant, MI 48858-0001
bob.a.howell@cmich.edu

Supplemental Material

Spectra for all Compounds

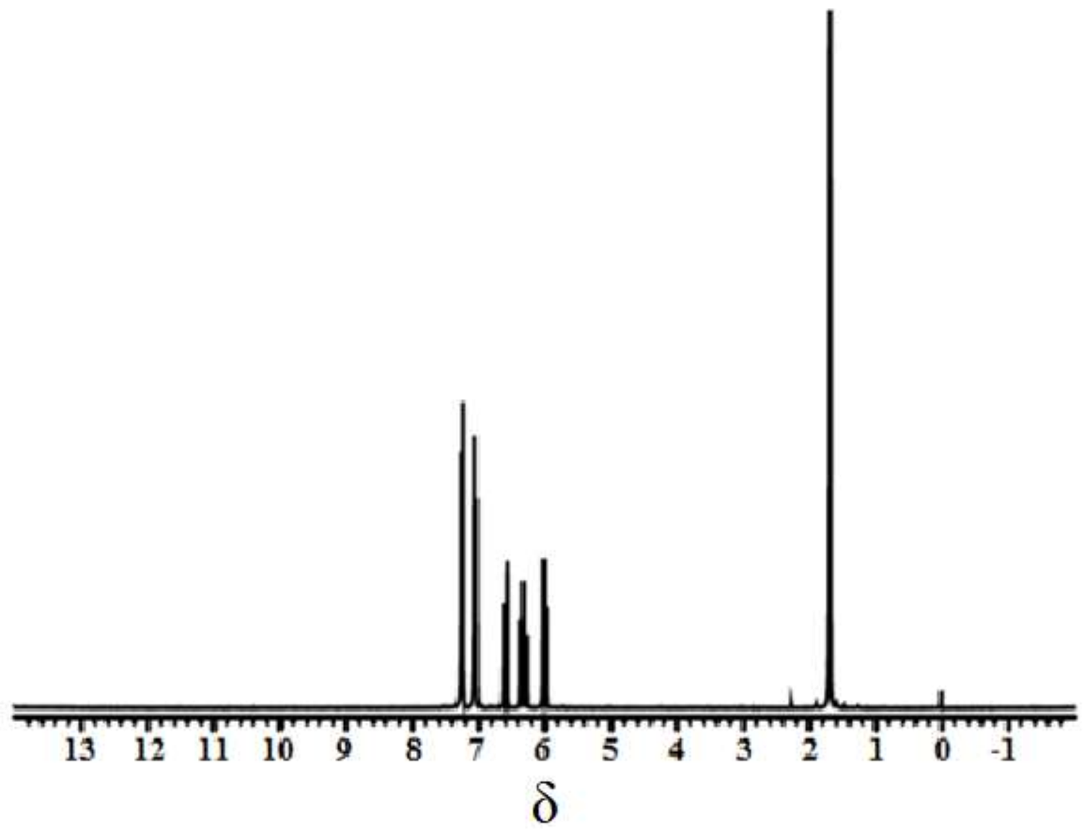

Figure S1. The ^{1}H NMR Spectrum for Bisphenol A Diacrylate (BPADA)

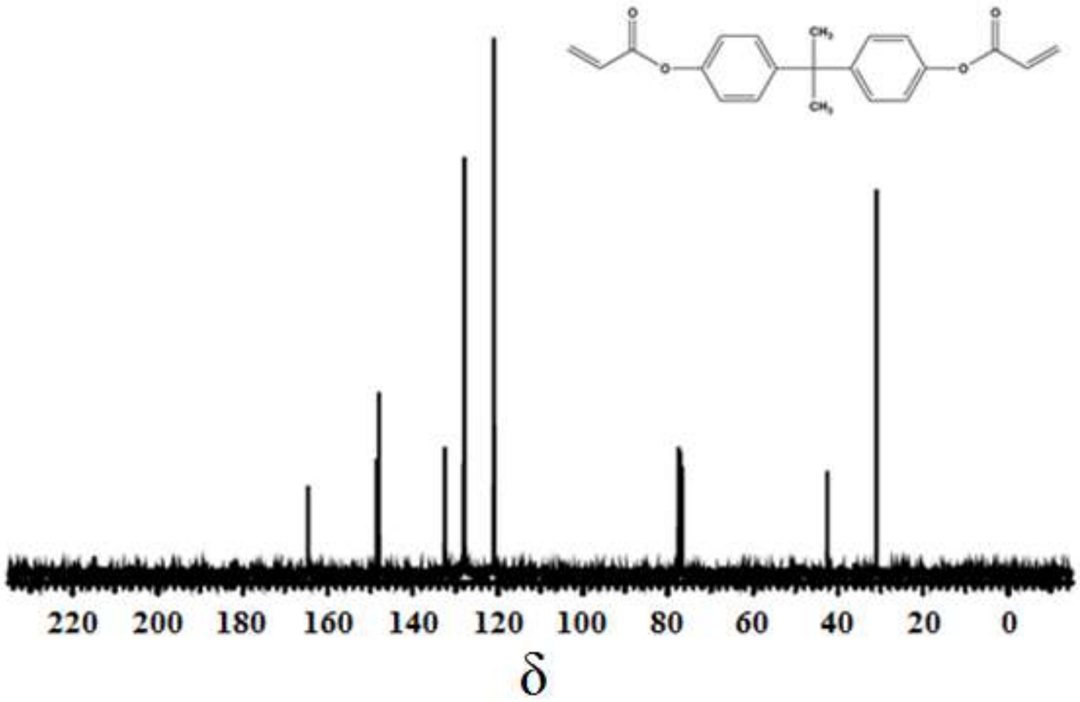

Figure S2. The ^{13}C NMR Spectrum for Bisphenol A Diacrylate (BPADA)

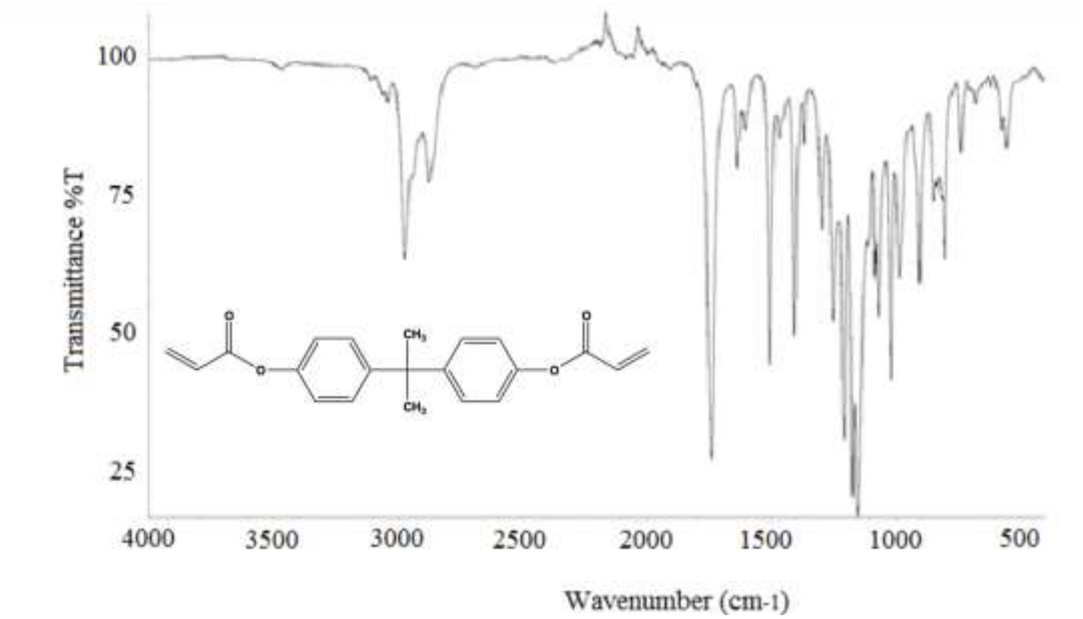

Figure S3. Infrared Spectrum of Bisphenol A Diacrylate (BPADA)

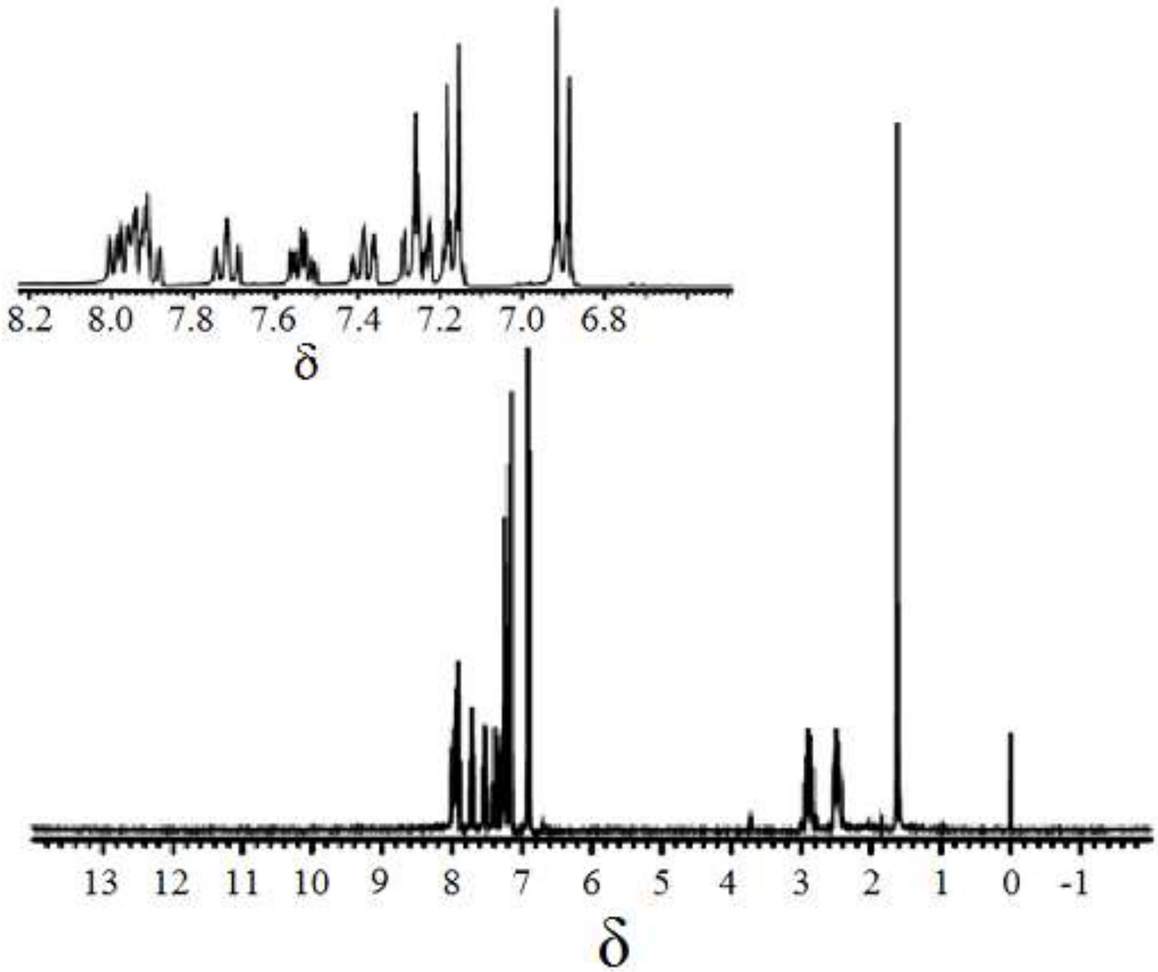

Figure S4. The ^{1}H NMR Spectrum of 2,2-Di[4-(3-dopylpropanoyl)phenyl]propane (DDPP)

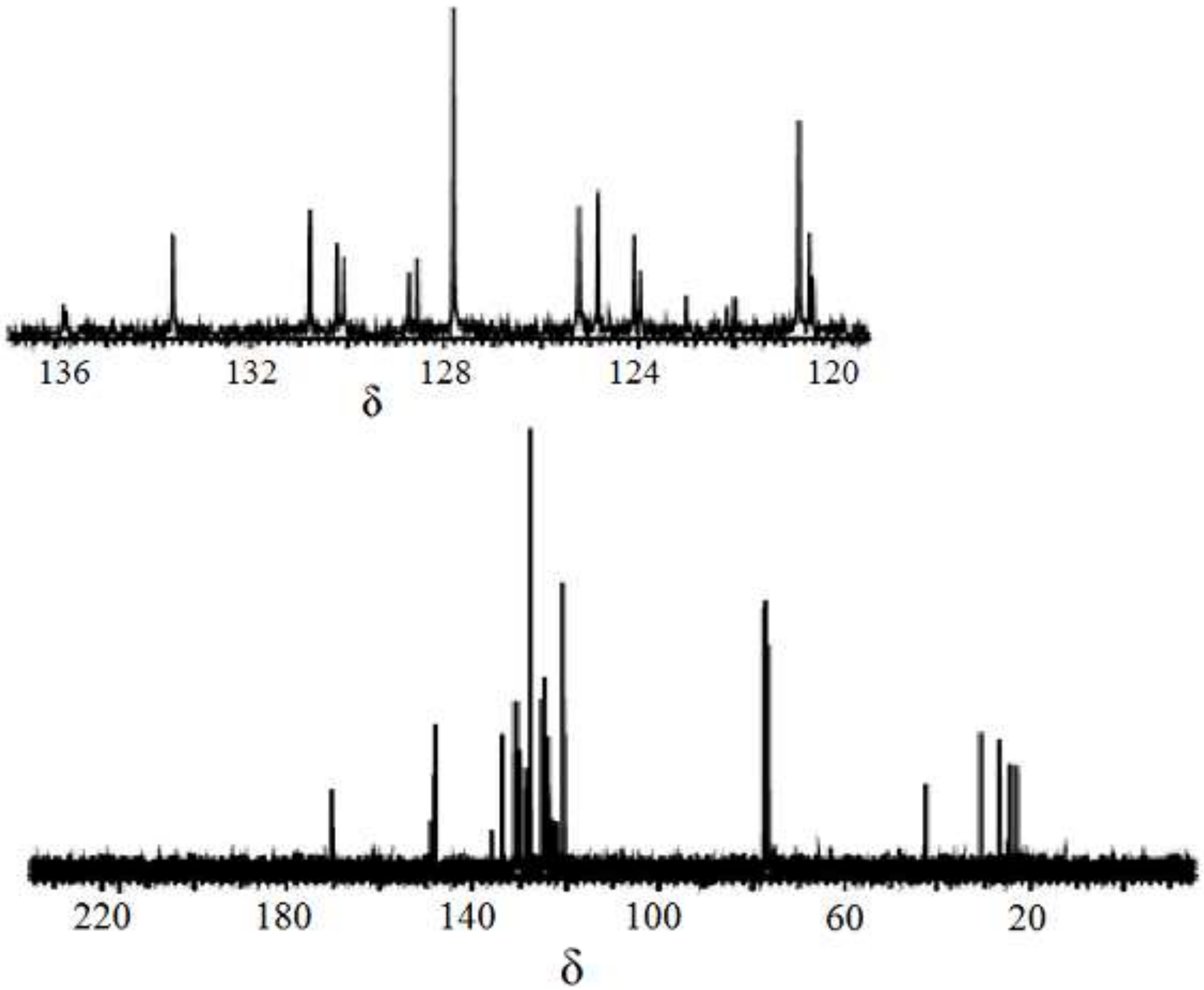

Figure S5. The ^{13}C NMR Spectrum of 2,2-Di[4-(3-dopylpropanoyl)phenyl]propane (DDPP)

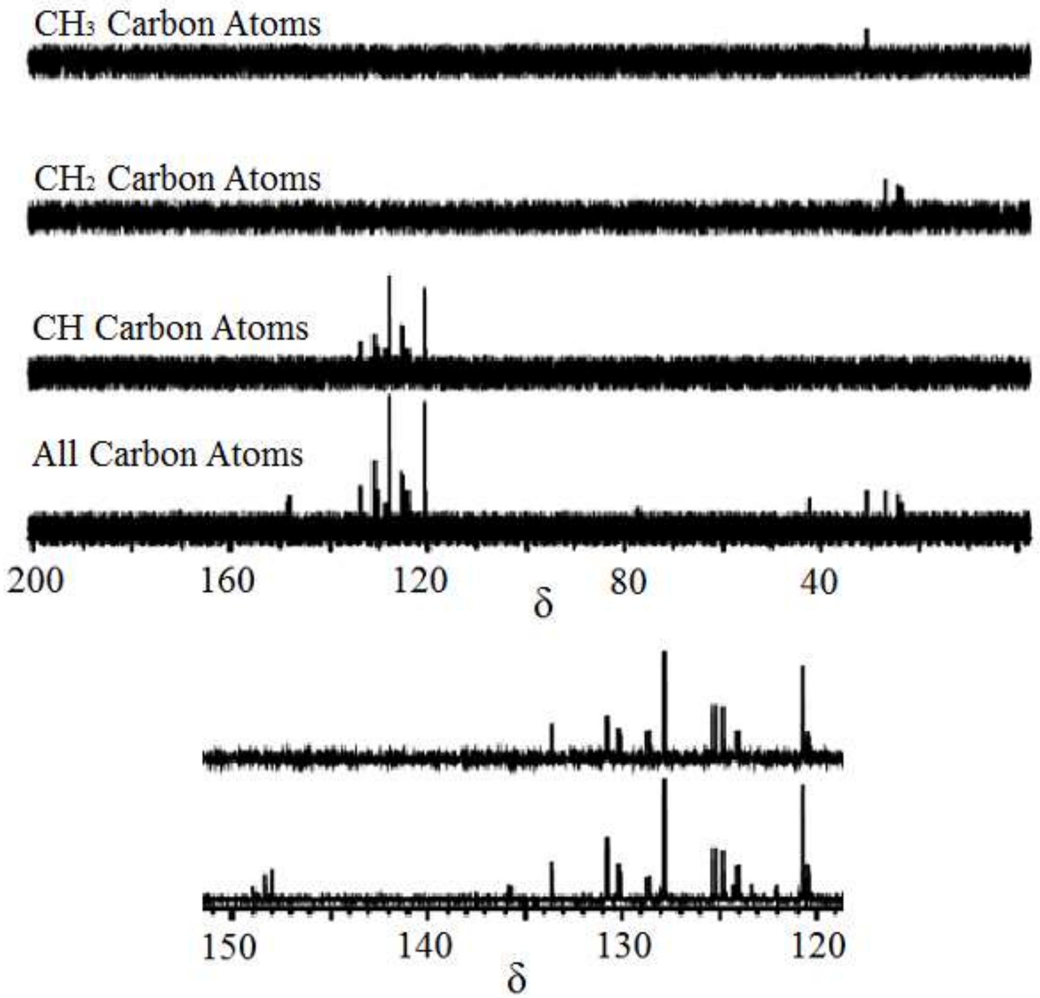

Figure S6. The DEPT NMR Spectrum of 2,2-Di[4-(3-dopylpropanoyl)phenyl]propane (DDPP)

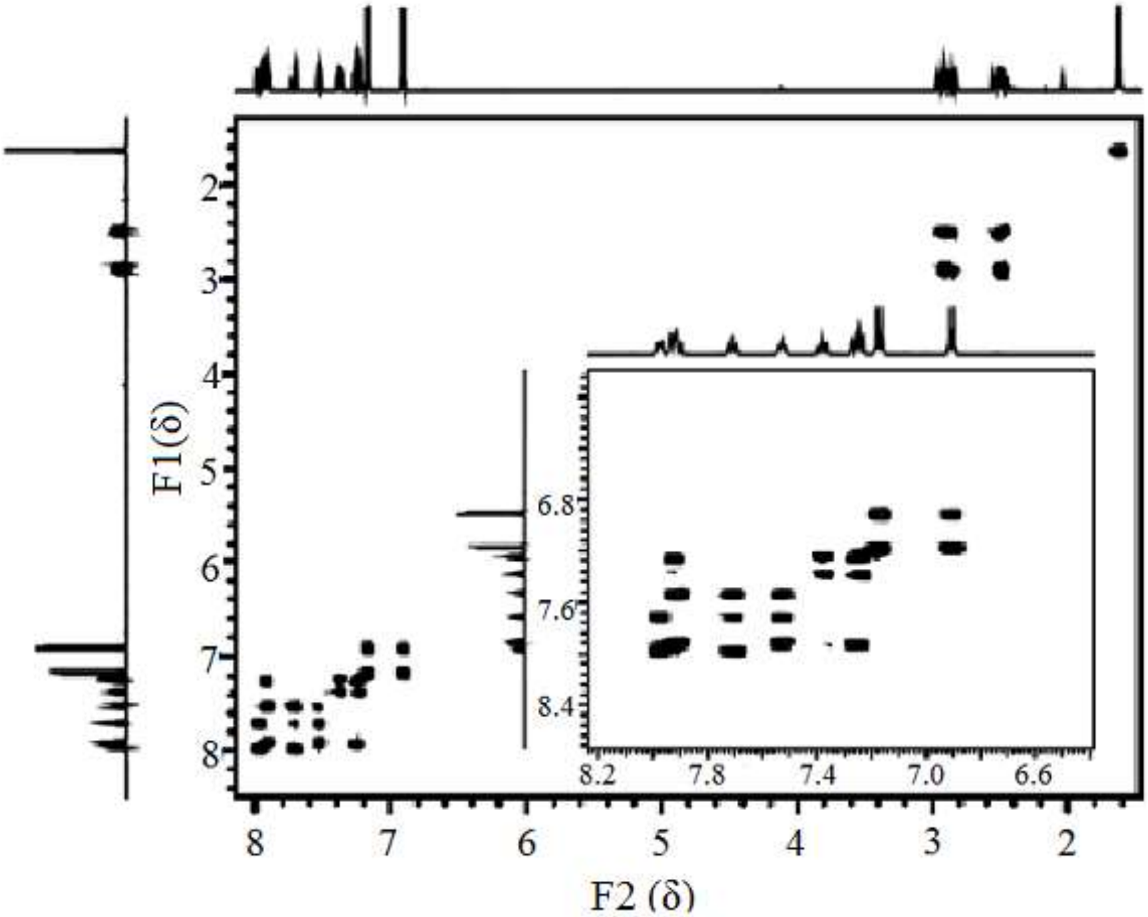

Figure S7. The gCOSY NMR Spectrum of 2,2-Di[4-(3-dopylpropanoyl)phenyl]propane (DDPP)

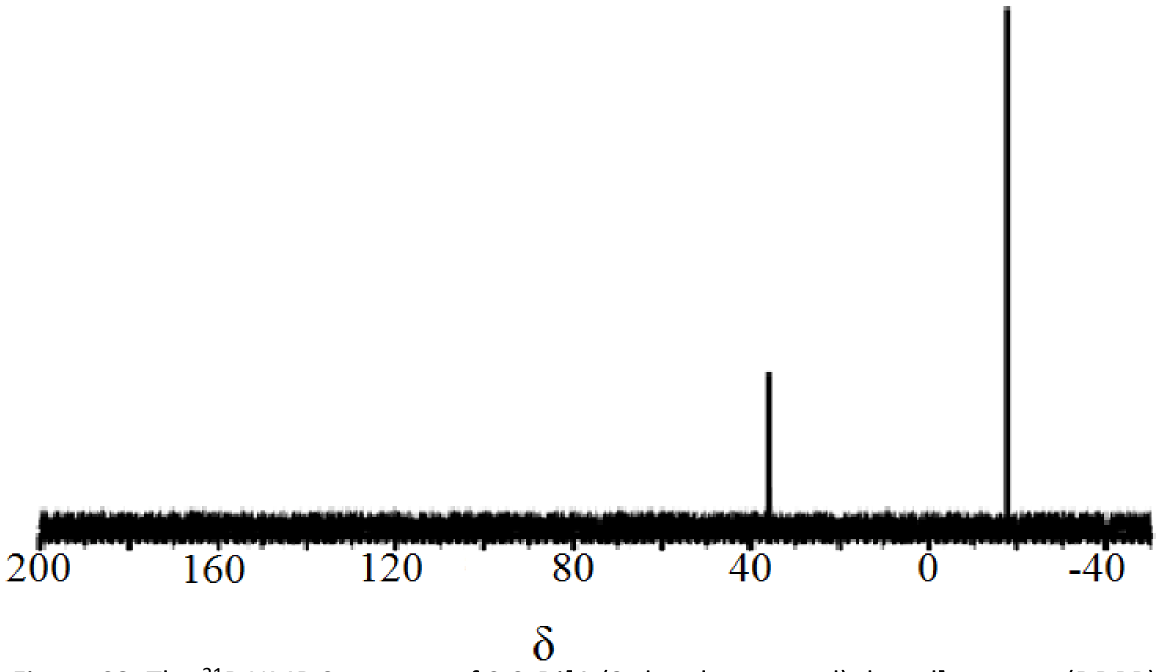

Figure S8. The ^{31}P NMR Spectrum of 2,2-Di[4-(3-dopylpropanoyl)phenyl]propane (DDPP)

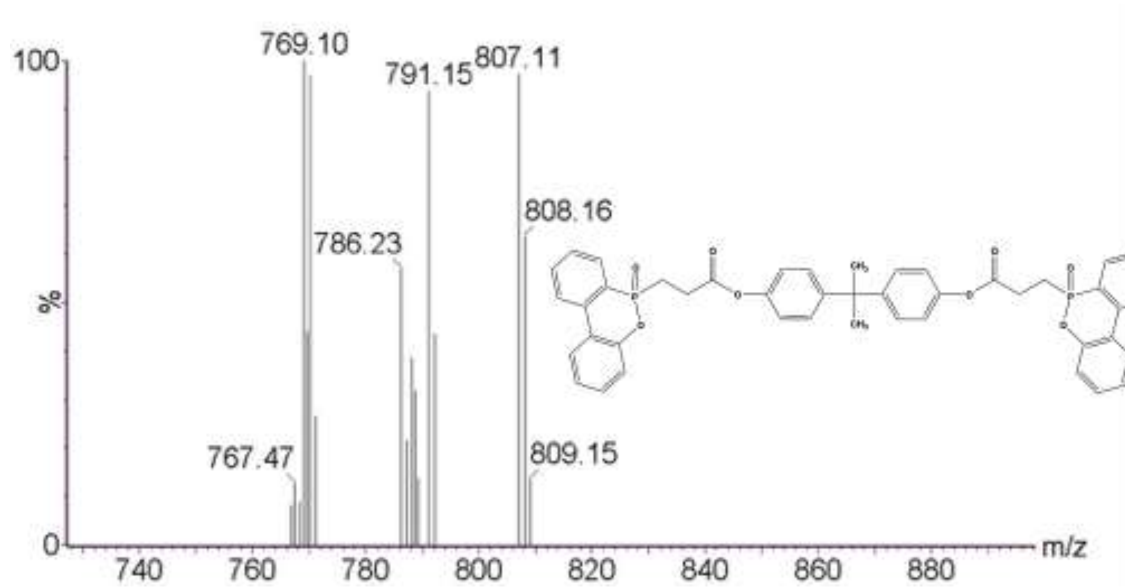

Figure S9. ESI Mass Spectrum of 2,2-Di[4-(3-dopylpropanoyl)phenyl]propane (DDPP)

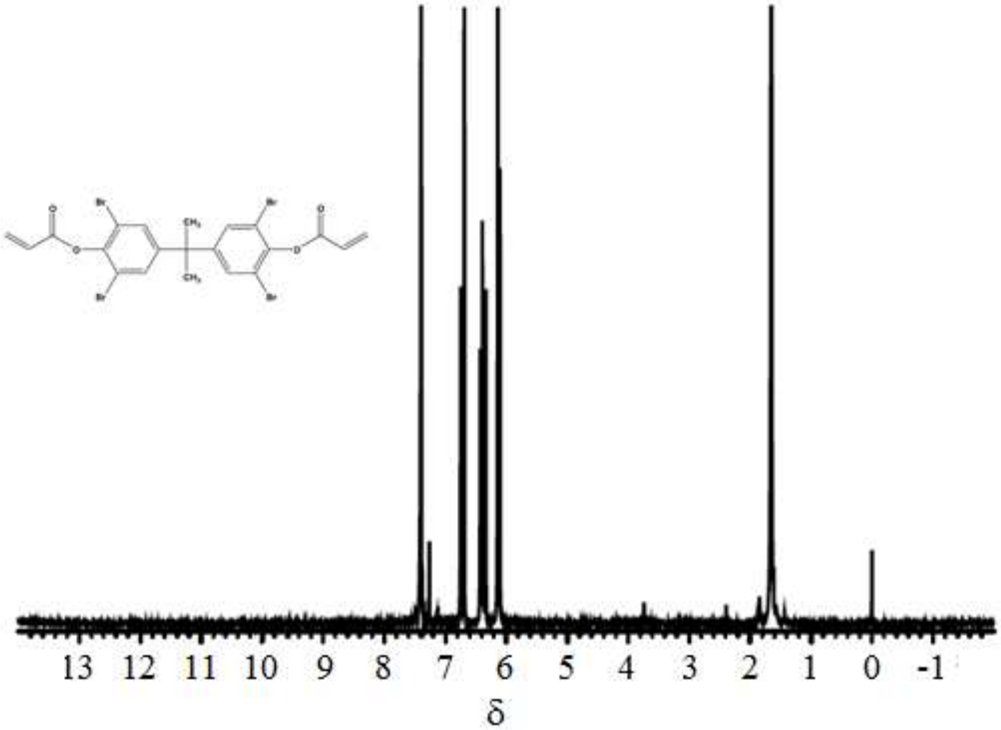

Figure S10. The ^{1}H NMR Spectrum for Tetrabromobisphenol A Diacrylate (BBPADA)

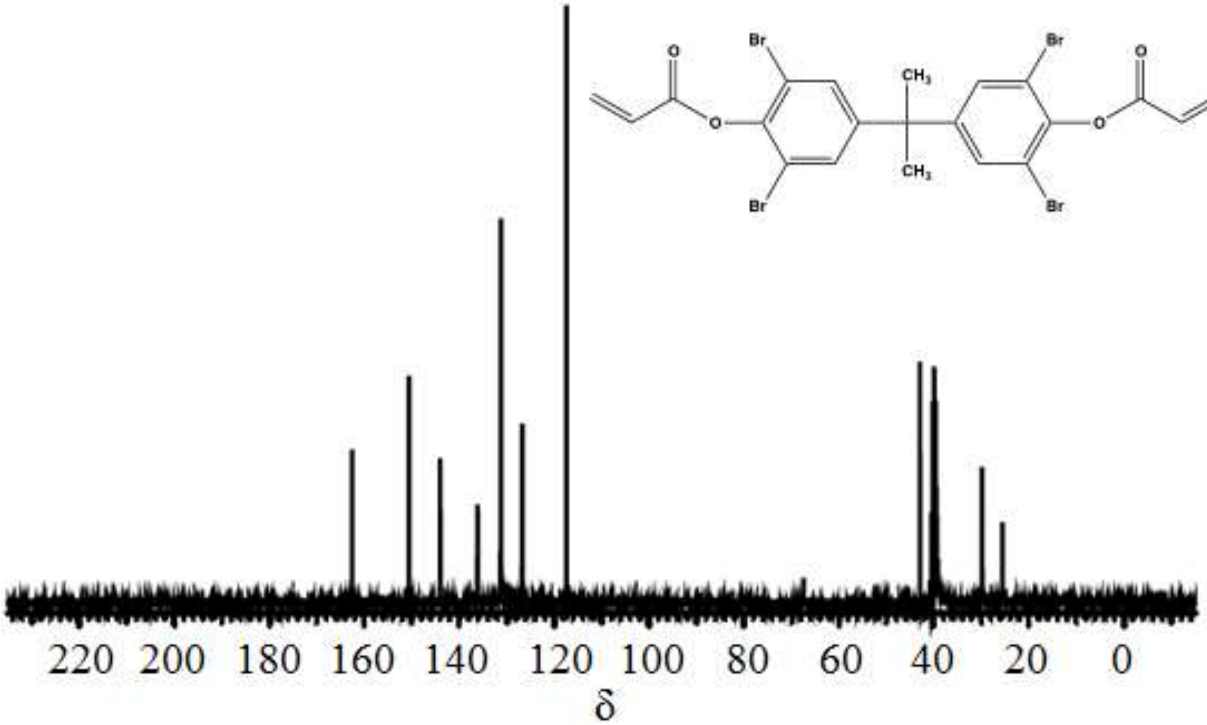

Figure S11. The ^{13}C NMR Spectrum for Tetrabromobisphenol A Diacrylate (BBPADA)

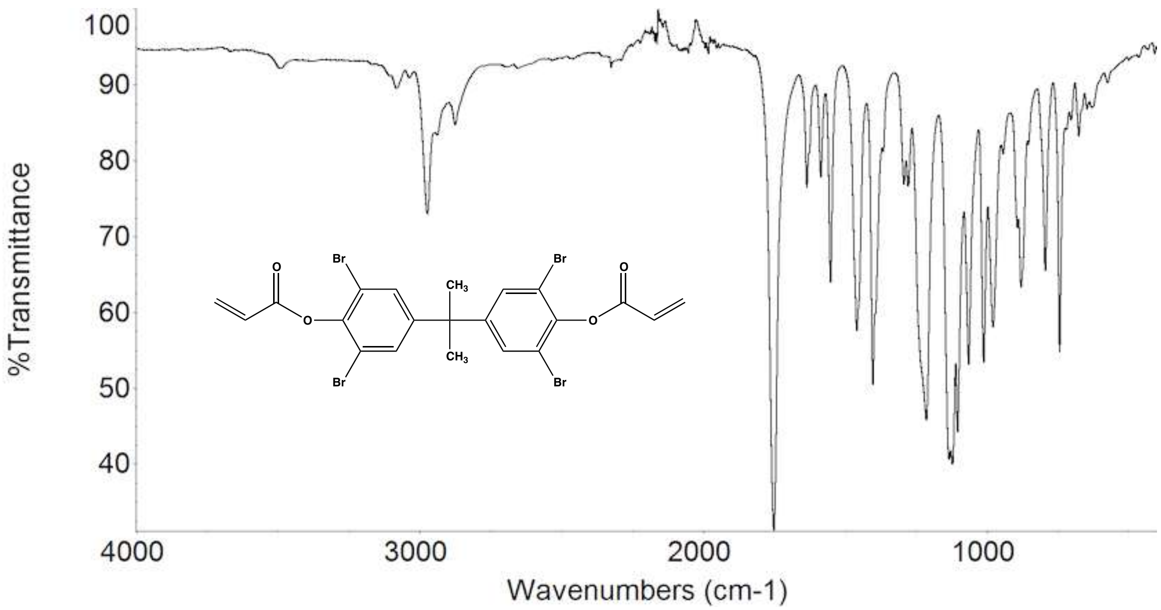

Figure S12. The Infrared Spectrum of of Tetrabromobisphenol A Diacrylate (BBPADA)

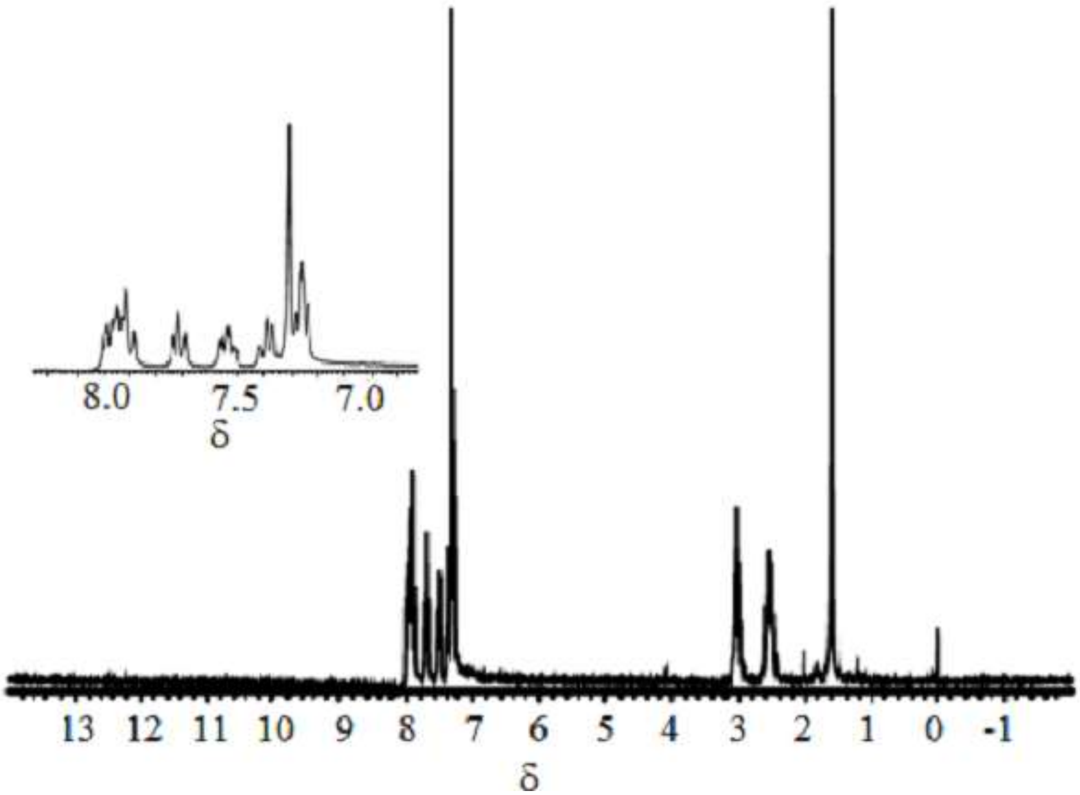

Figure S13. The ^{1}H NMR Spectrum 2,2-Di[3,5-dibromo-4-(3-dopylpropanoyl)phenyl]propane (DBDPP)

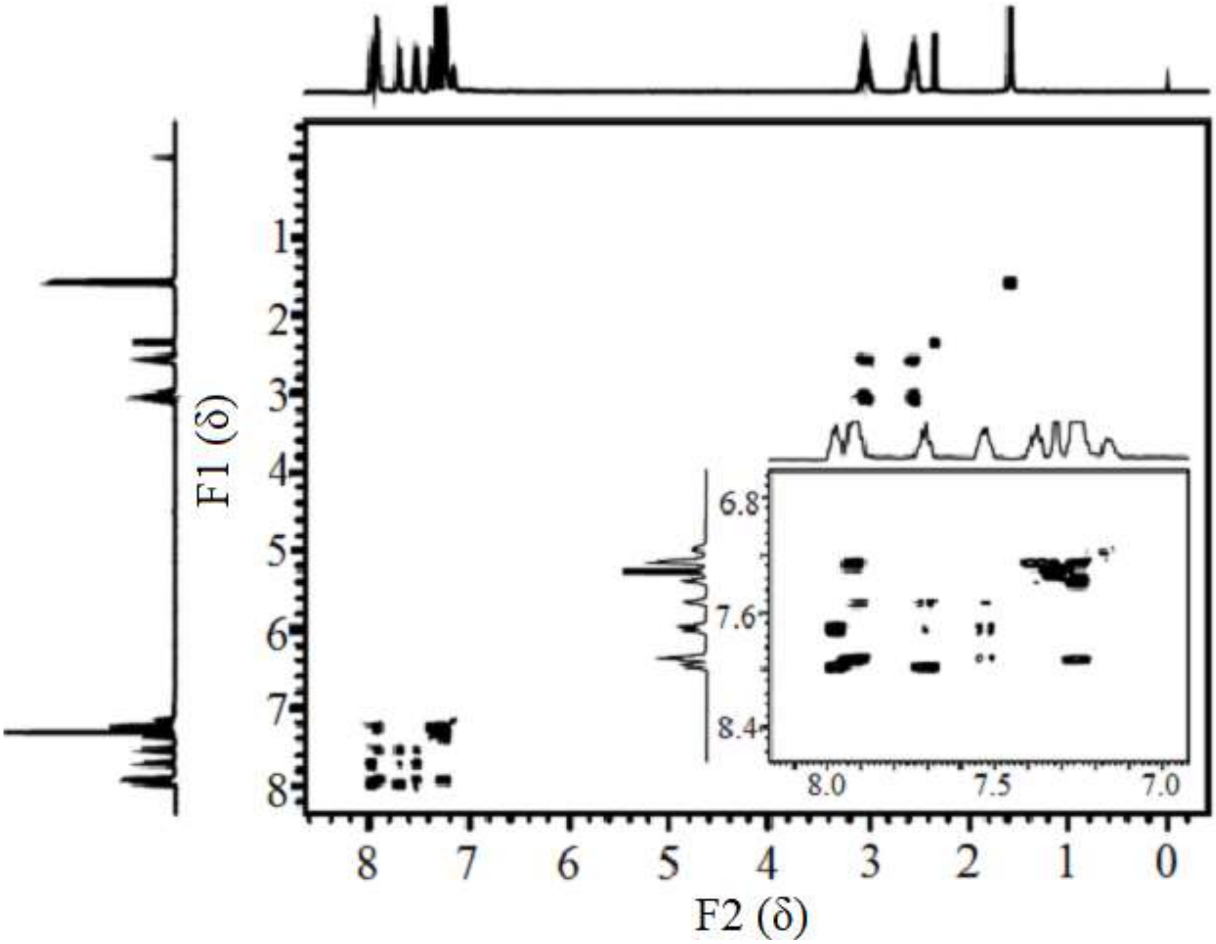

Figure S14. The gCOSY NMR Spectrum for 2,2-Di[3,5-dibromo-4-(3-dopylpropanoyl)phenyl]propane (DBDPP).

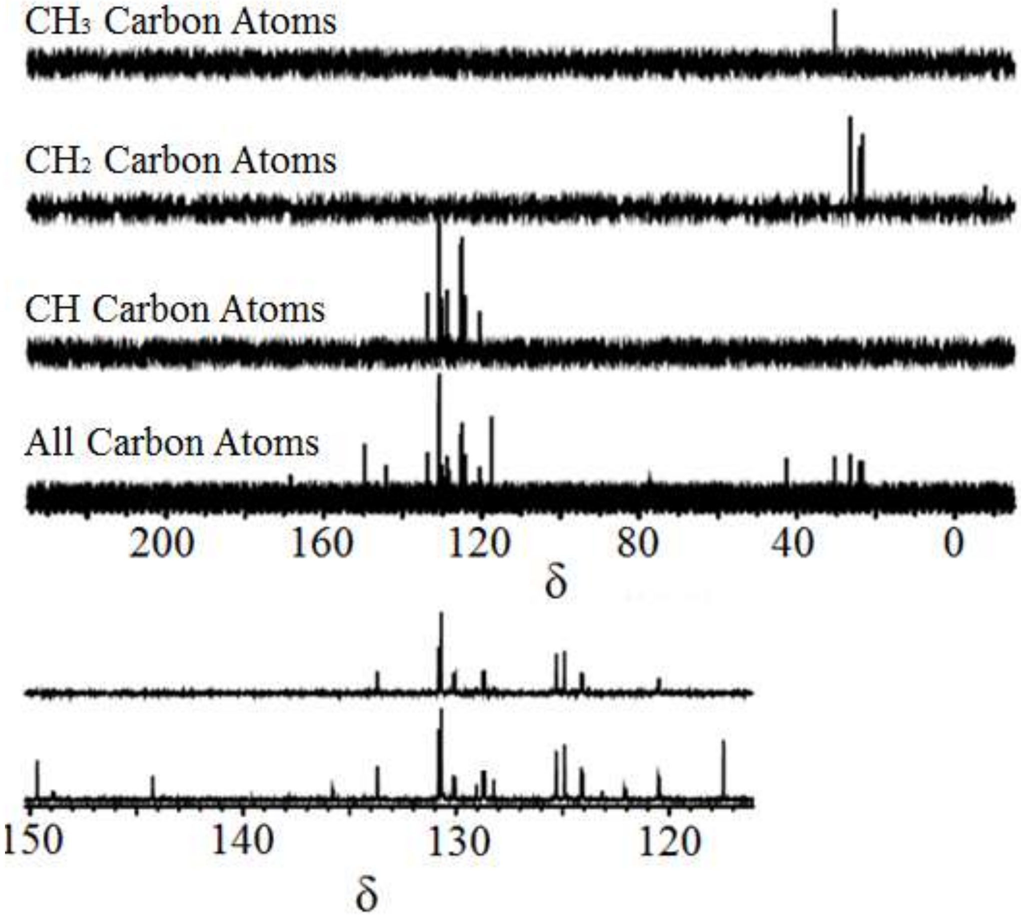

Figure S15. The DEPT NMR Spectrum for 2,2-Di[3,5-dibromo-4-(3-dopylpropanoyl)phenyl]propane (DBDPP).

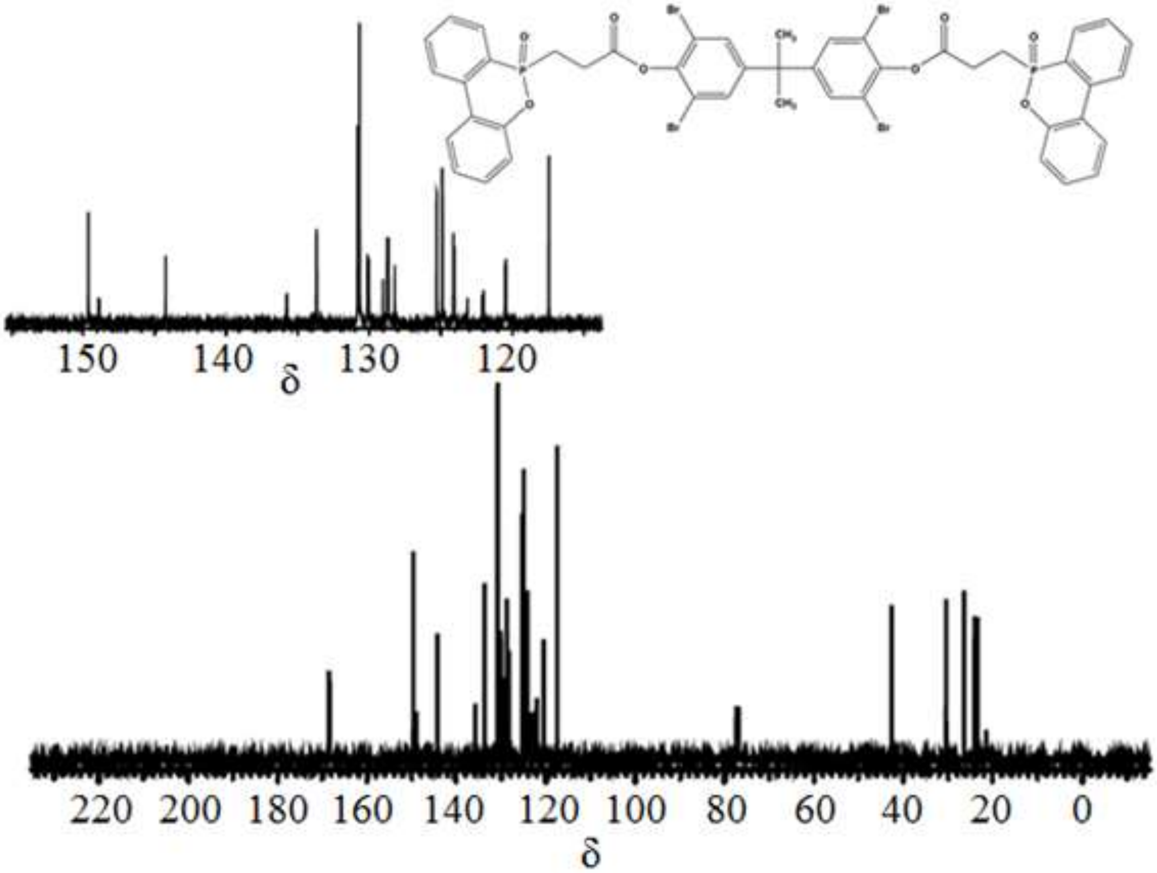

Figure S16. The ^{13}C NMR Spectrum for 2,2-Di[3,5-dibromo-4-(3-dopylpropanoyl)phenyl]propane (DBDPP).

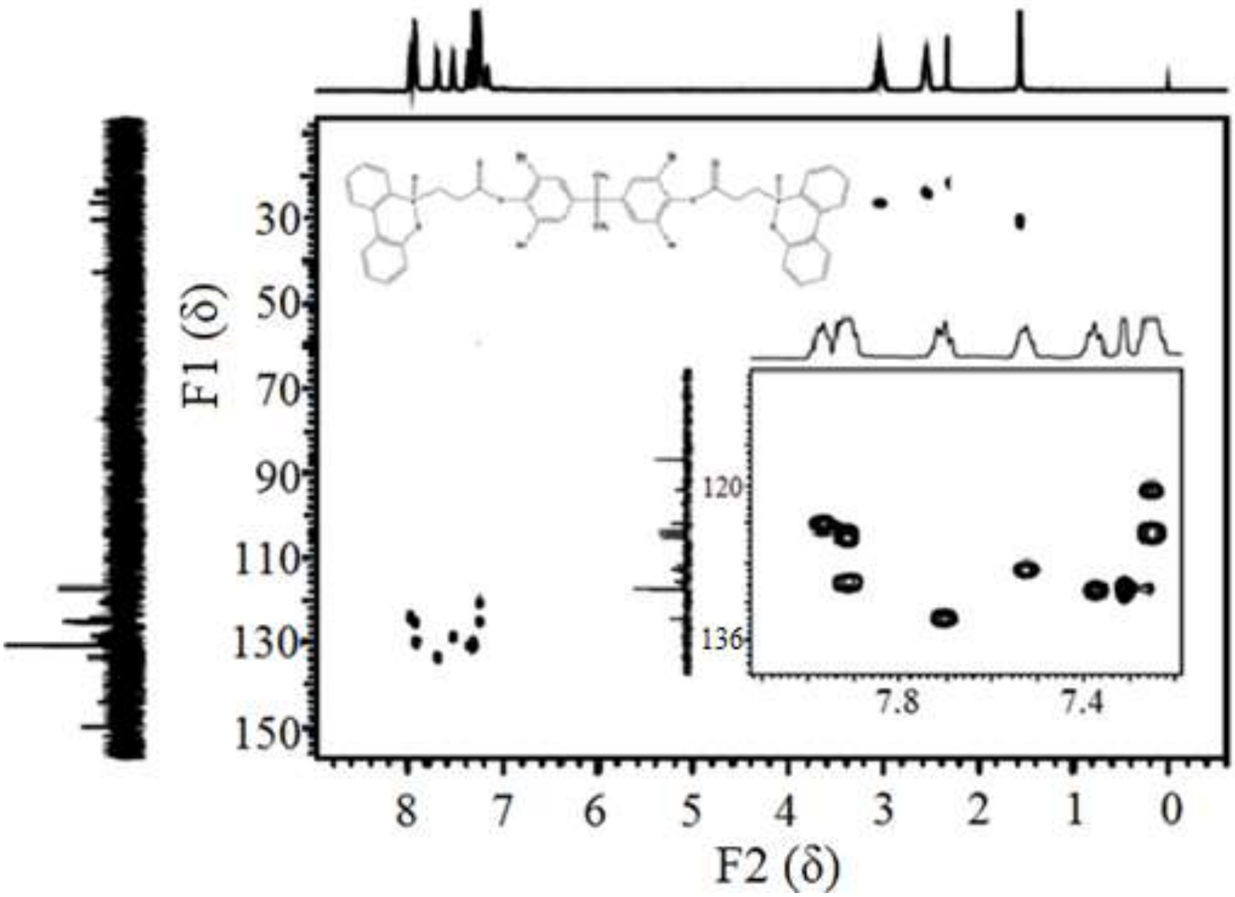

Figure S17. The HSQCAD NMR Spectrum for 2,2-Di[3,5-dibromo-4-(3-dopylpropanoyl)phenyl]propane (DBDPP).

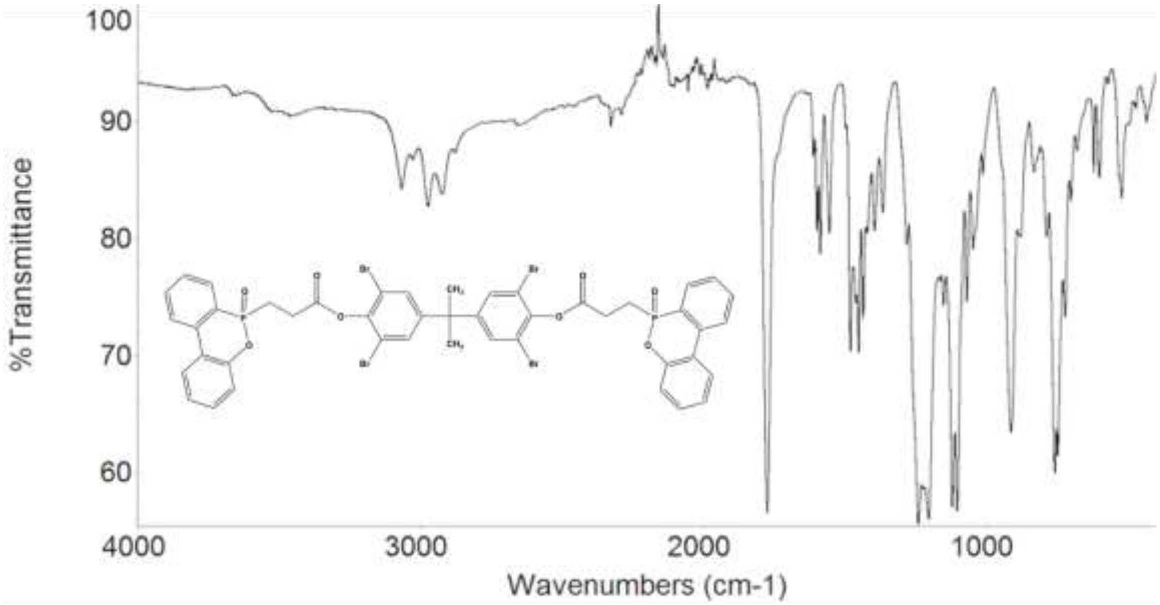

Figure S18. The Infrared Spectrum of 2,2-Di[3,5-dibromo-4-(3-dopylpropanoyl)phenyl]propane (DBDPP).

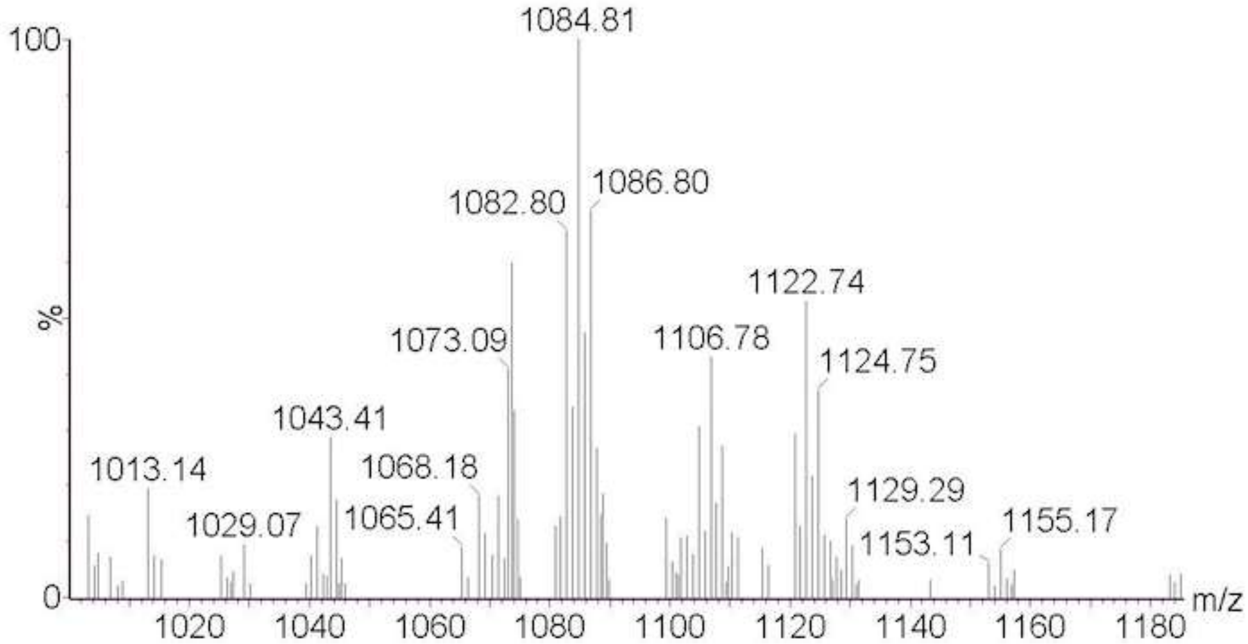

Figure S19. The ESI Mass Spectrum of 2,2-Di[3,5-dibromo-4-(3-dopylpropanoyl)phenyl]propane (DBDPP).

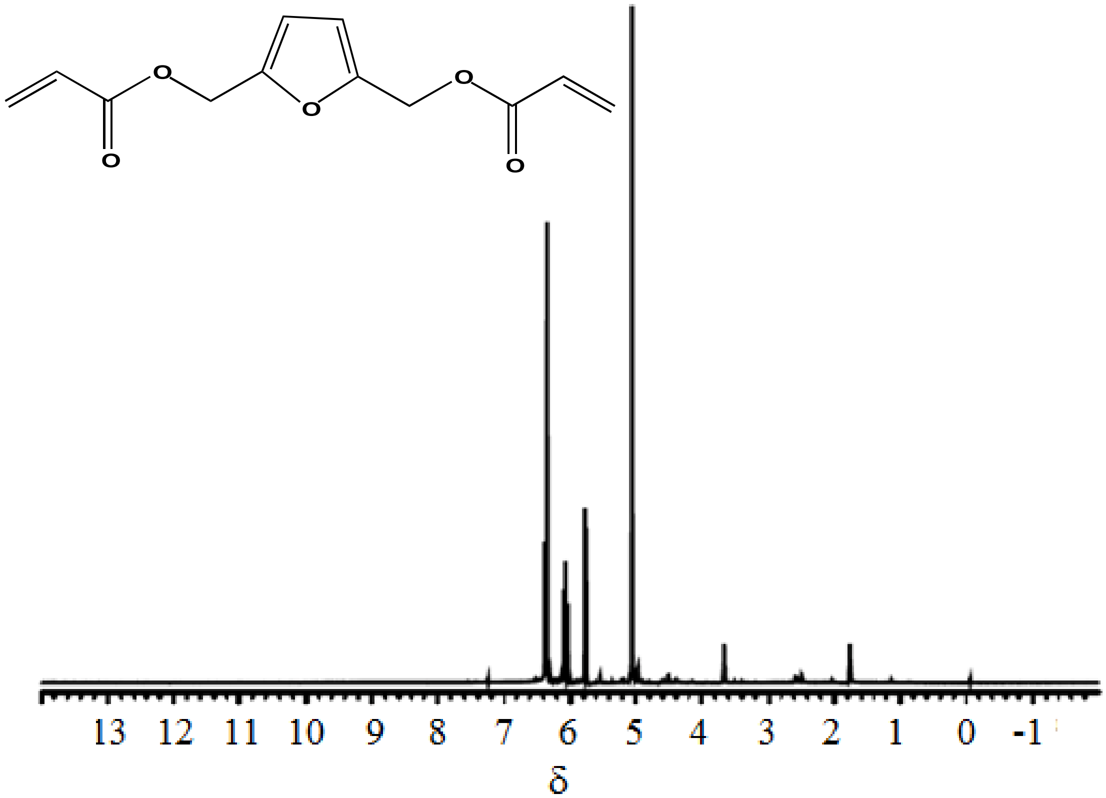

Figure S20. The ^{1}H NMR Spectrum for 2,5-*bis*-(Hydroxymethyl)furan Diacrylate (BHFDA)

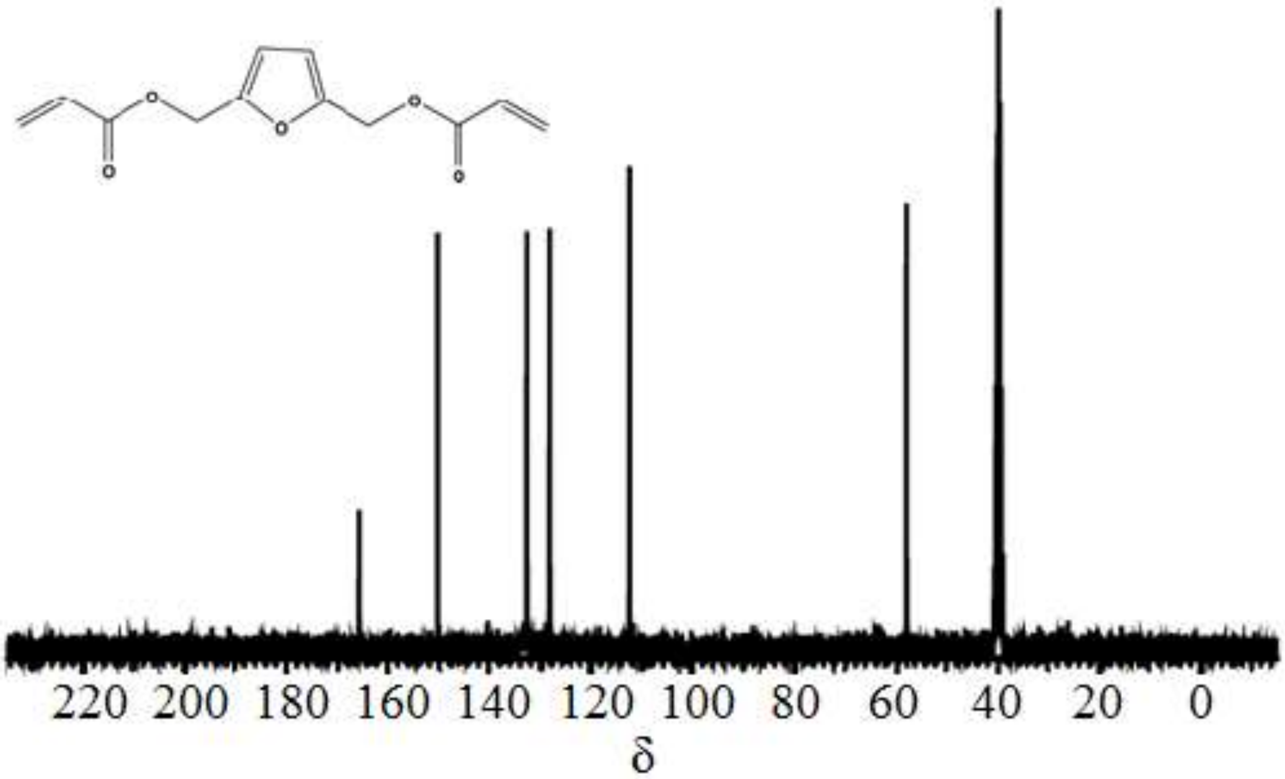

Figure S21. The ^{13}C NMR Spectrum for 2,5-*bis*-(Hydroxymethyl)furan Diacrylate (BHFDA)

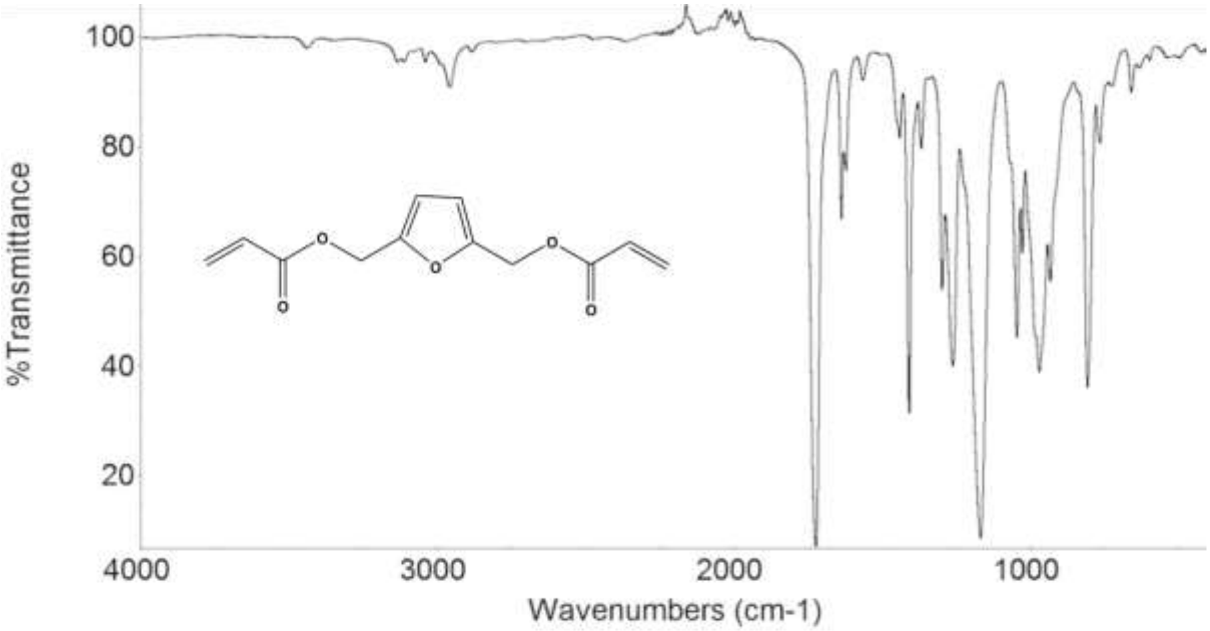

Figure S22. The Infrared Spectrum for 2,5-*bis*-(Hydroxymethyl)furan Diacrylate (BHFDA)

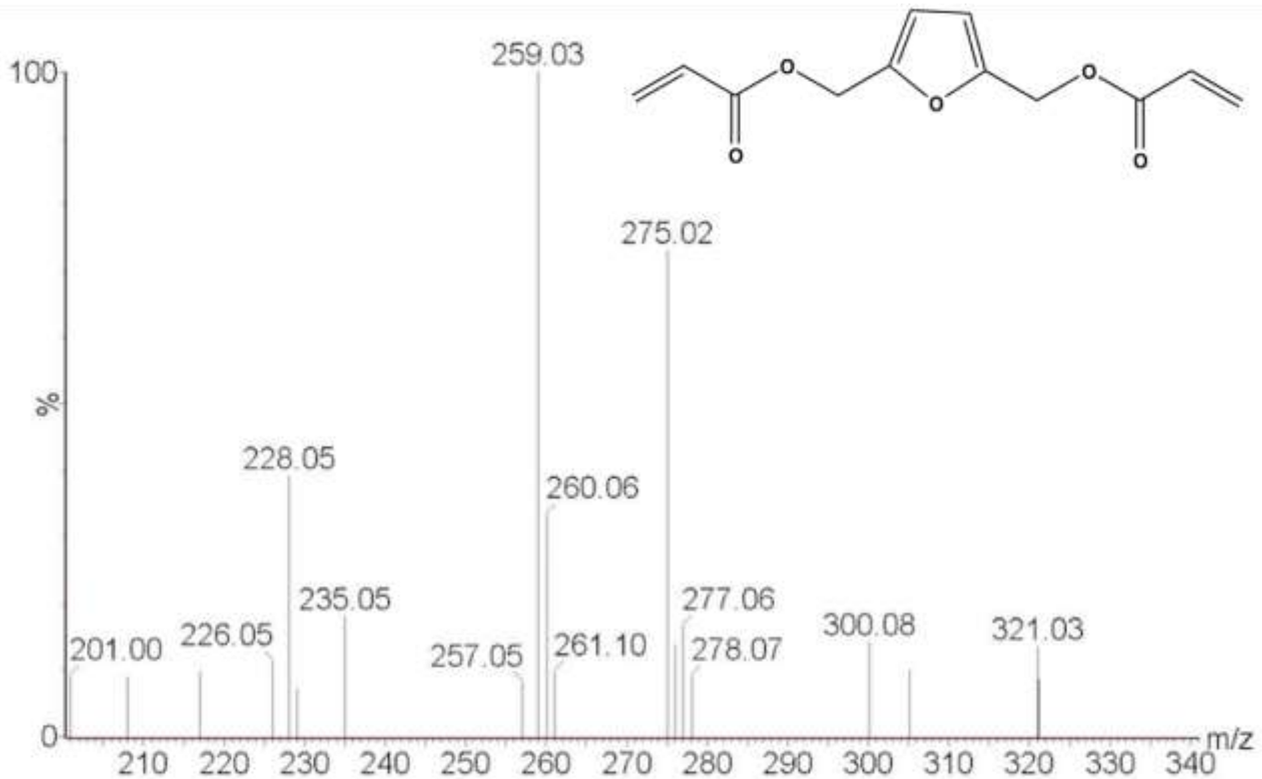

Figure S23. Mass Spectrum for 2,5-*bis*-(Hydroxymethyl)furan Diacrylate (BHFDA)

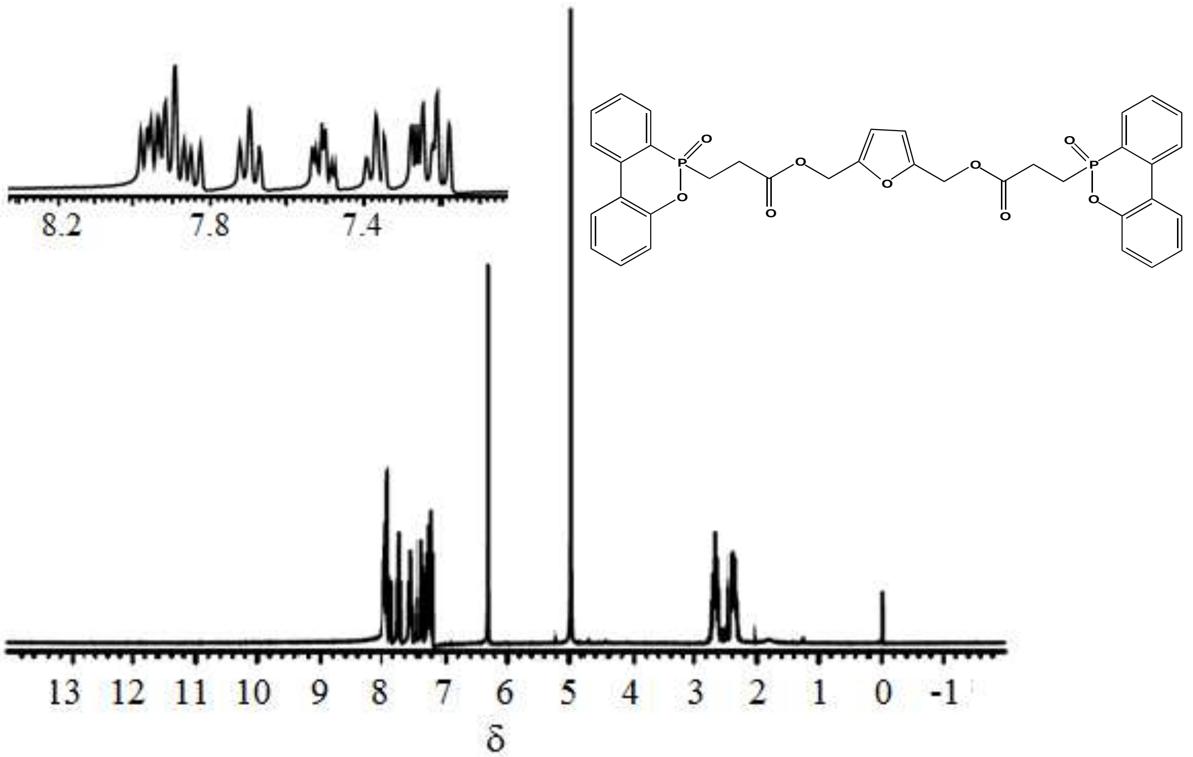

Figure S24. The ^{1}H NMR Spectrum for 2,5-Di[(3-dopylpropanoyl)methyl]furan (DDMF)

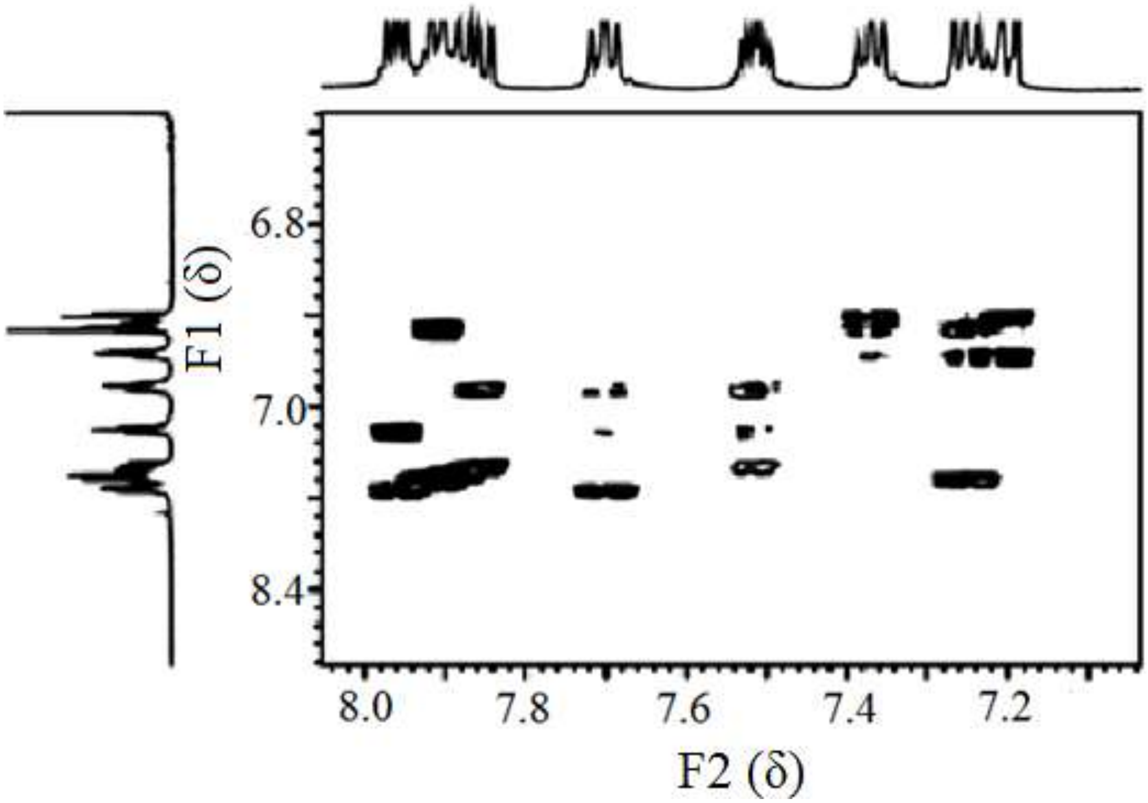

Figure S25. The gCOSY NMR Spectrum for 2,5-Di[(3-dopylpropanoyl)methyl]furan (DDMF)

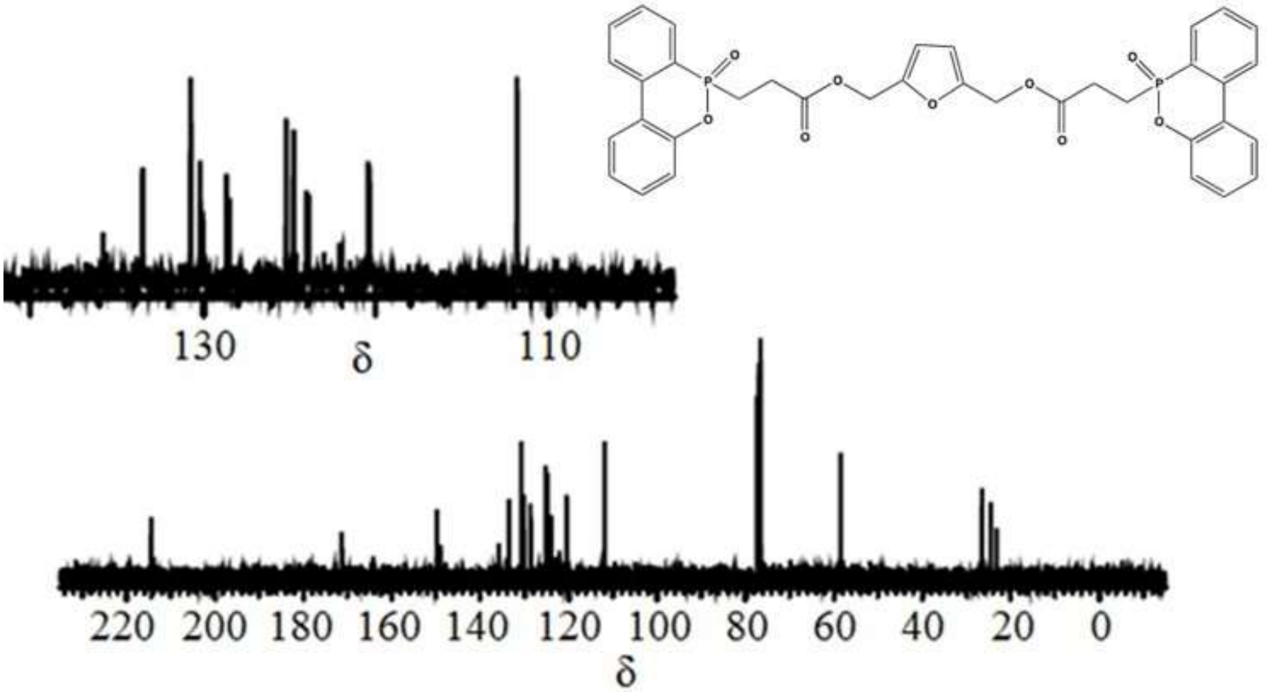

Figure S26. The ^{13}C NMR Spectrum for 2,5-Di[(3-dopylpropanoyl)methyl]furan (DDMF)

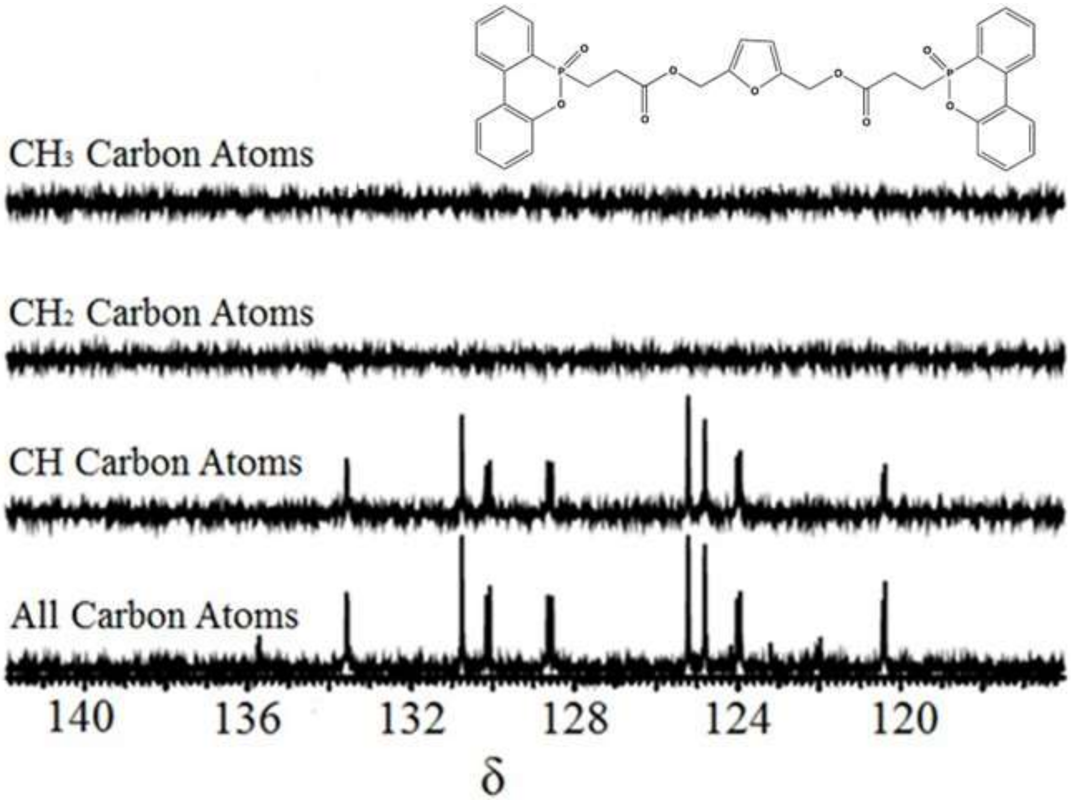

Figure S27. The DEPT NMR Spectrum for 2,5-Di[(3-dopylpropanoyl)methyl]furan (DDMF)

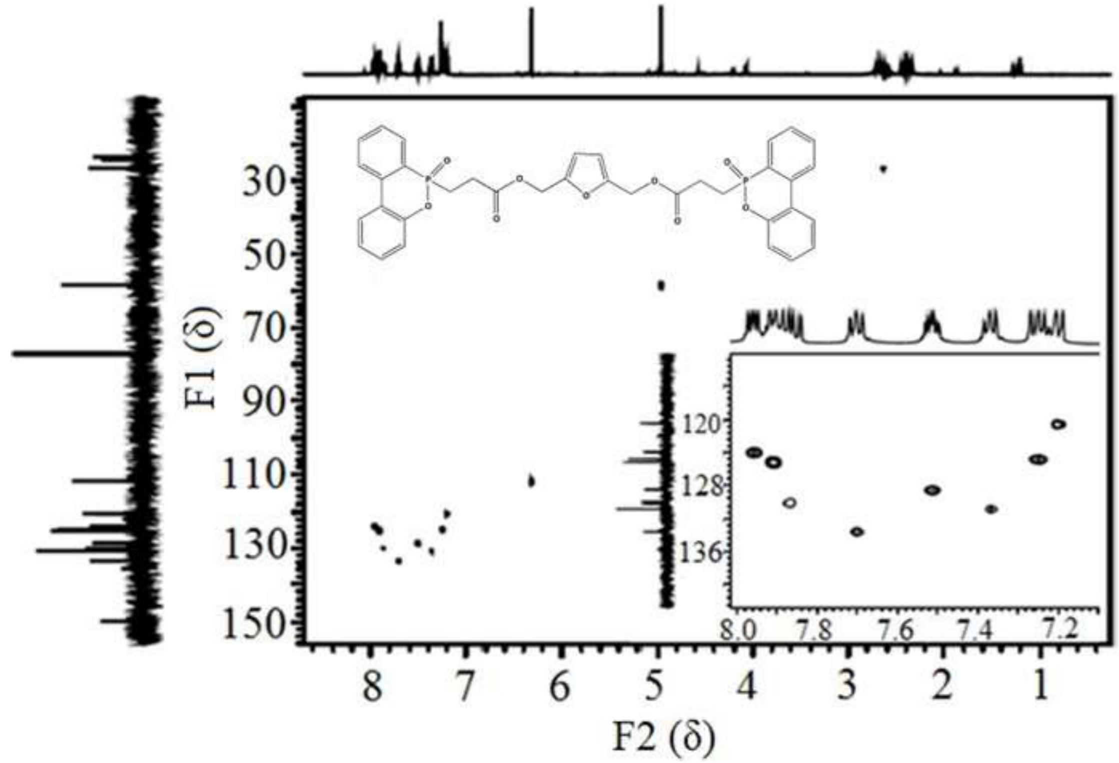

Figure S28. The HSQCDA NMR Spectrum for 2,5-Di[(3-dopylpropanoyl)methyl]furan (DDMF)

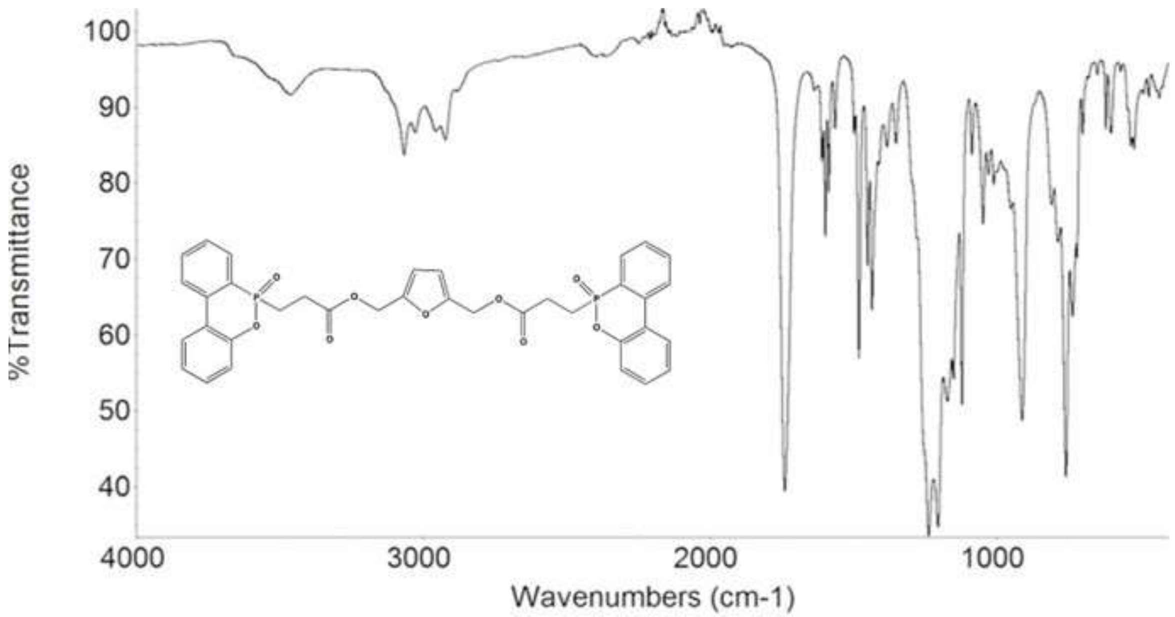

Figure S29. The Infrared Spectrum of 2,5-Di[(3-dopylpropanoyl)methyl]furan (DDMF)

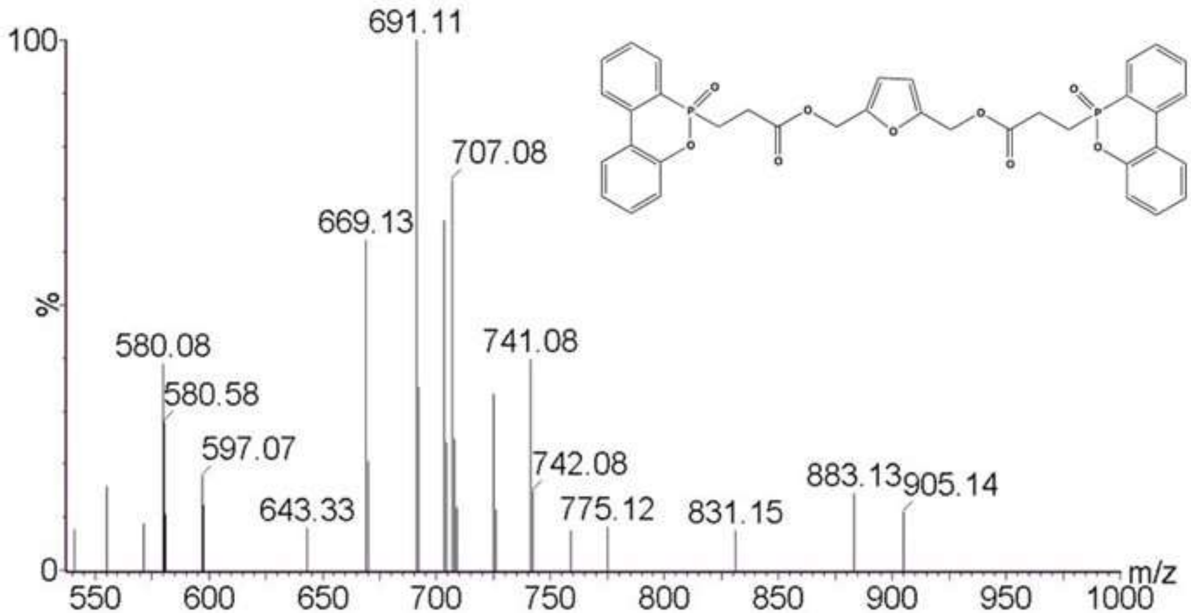

Figure S30. The ESI Mass Spectrum of 2,5-Di[(3-dopylpropanoyl)methyl]furan (DDMF)

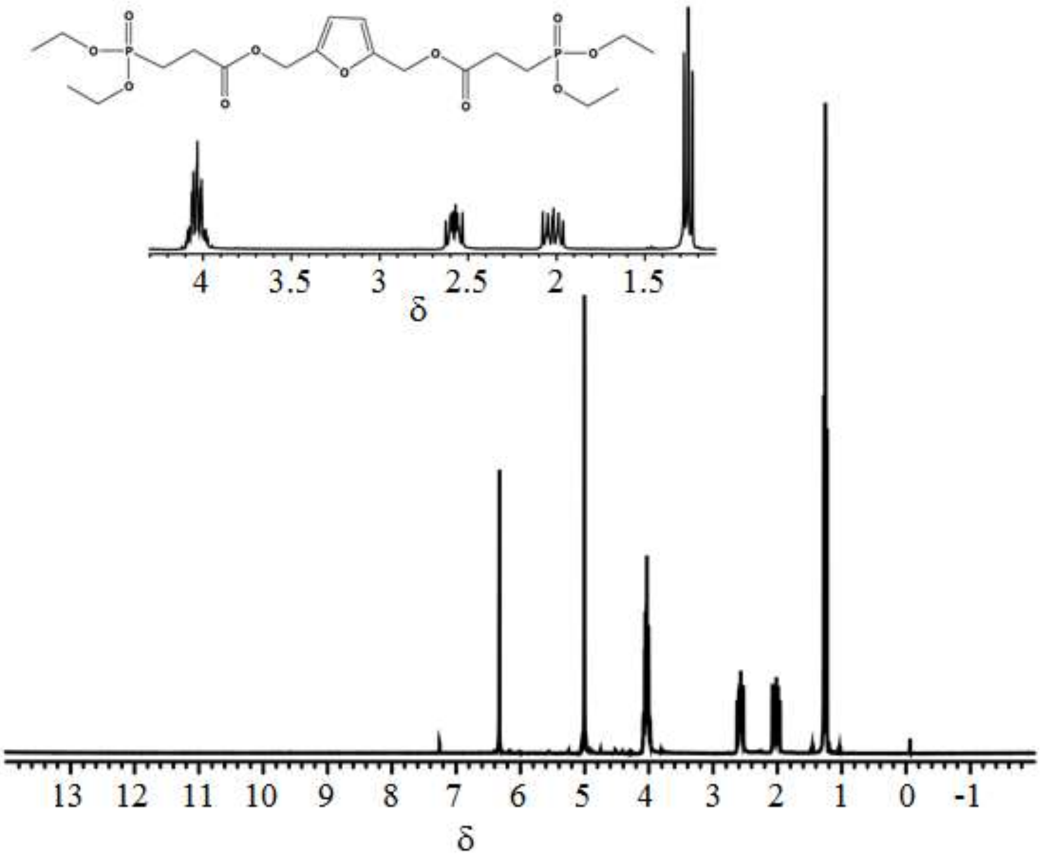

Figure S31. The ^{1}H NMR Spectrum for Di[(3-diethylphosphonatopropanoyl)methyl]furan (DEMF)

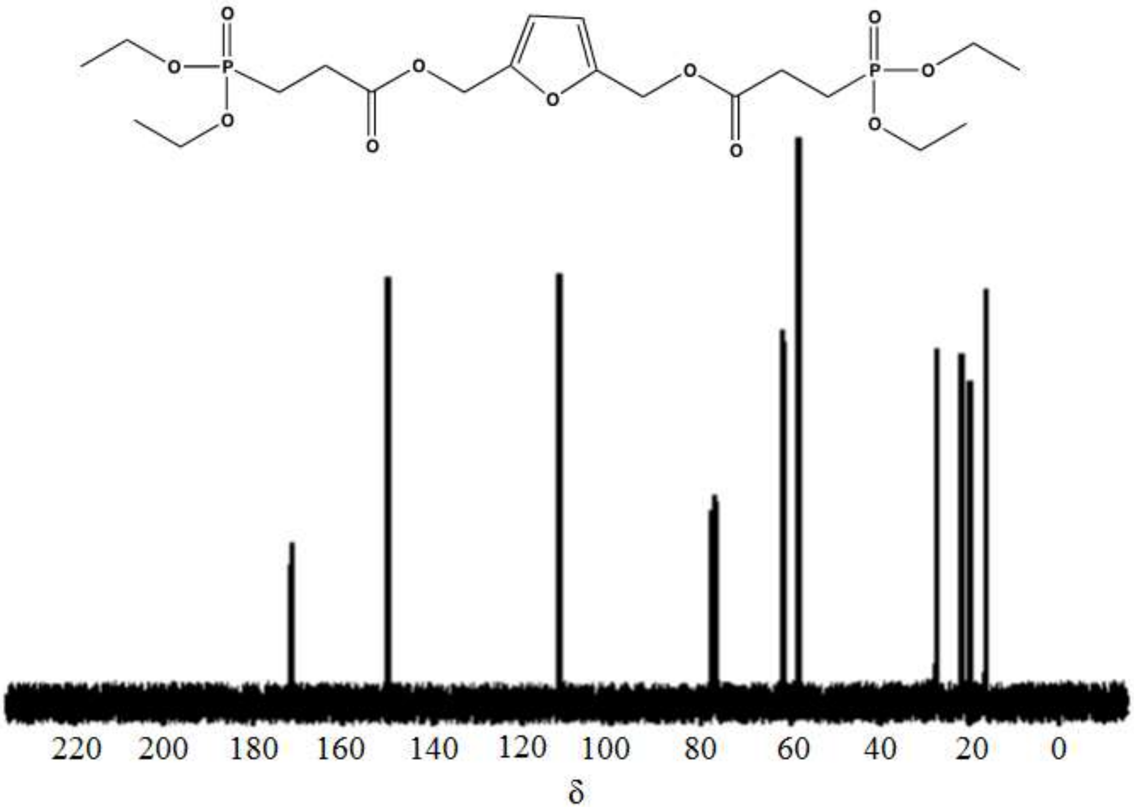

Figure S32. The ^{13}C NMR Spectrum for Di[(3-diethylphosphonatopropanoyl)methyl]furan (DEMF)

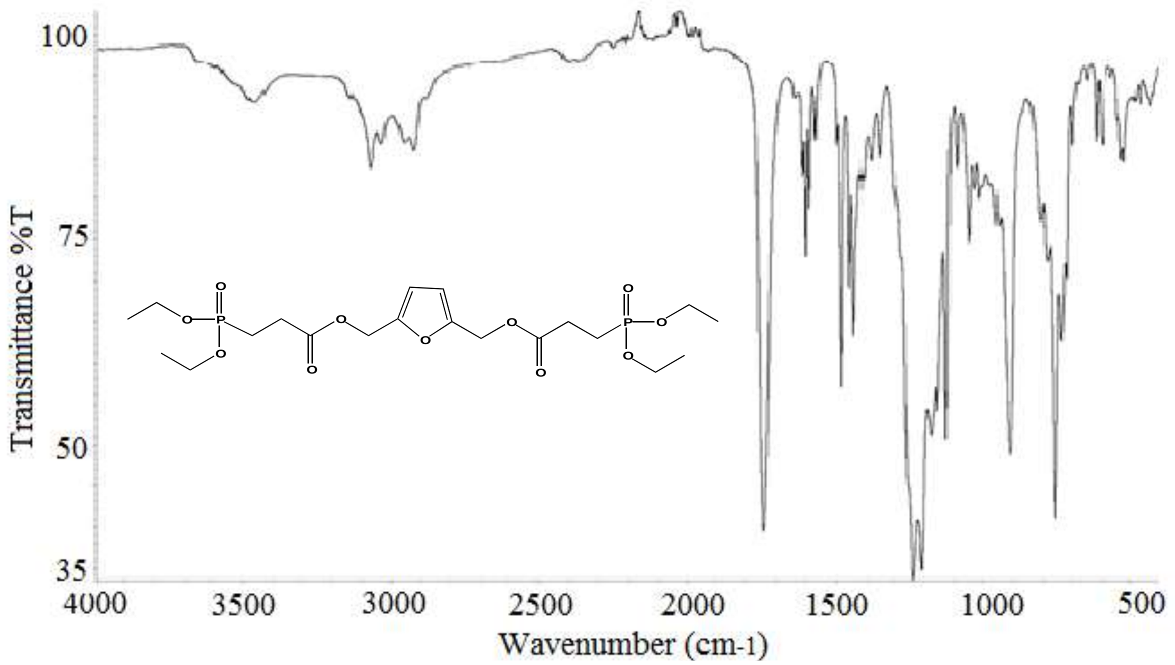

Figure S33. The Infrared Spectrum of Di[(3-diethylphosphonatopropanoyl)methyl]furan (DEMF)

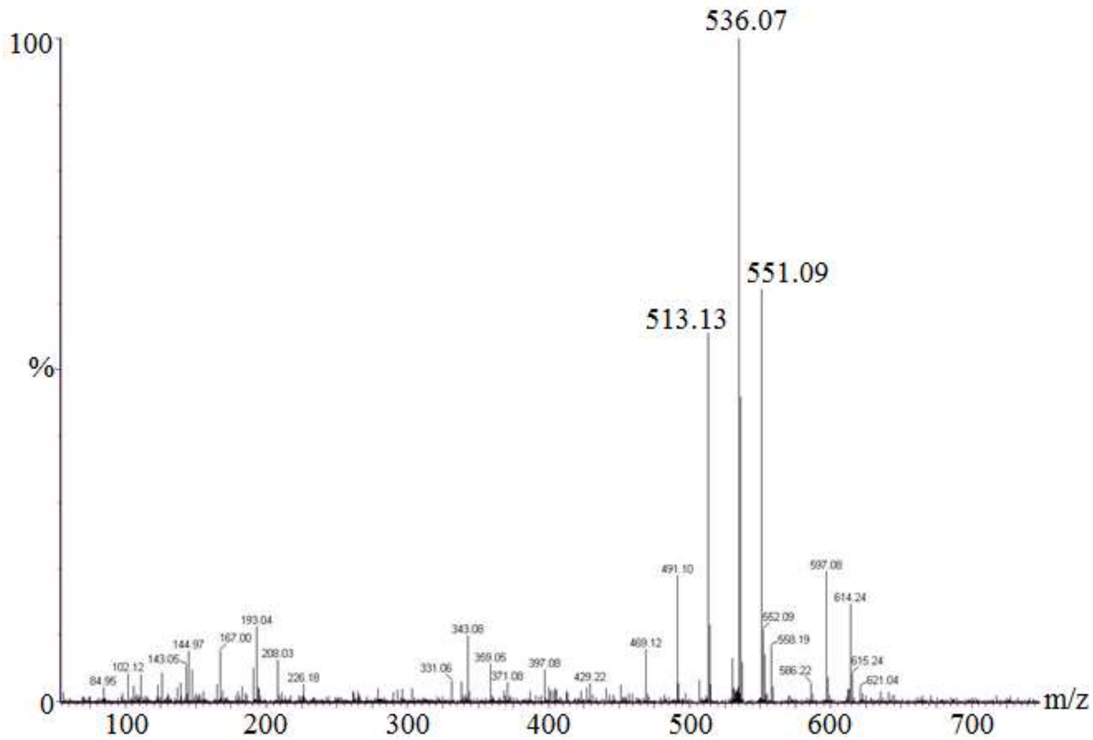

Figure S34. The ESI Mass Spectrum of Di[(3-diethylphosphonatopropanoyl)methyl]furan (DEMF)

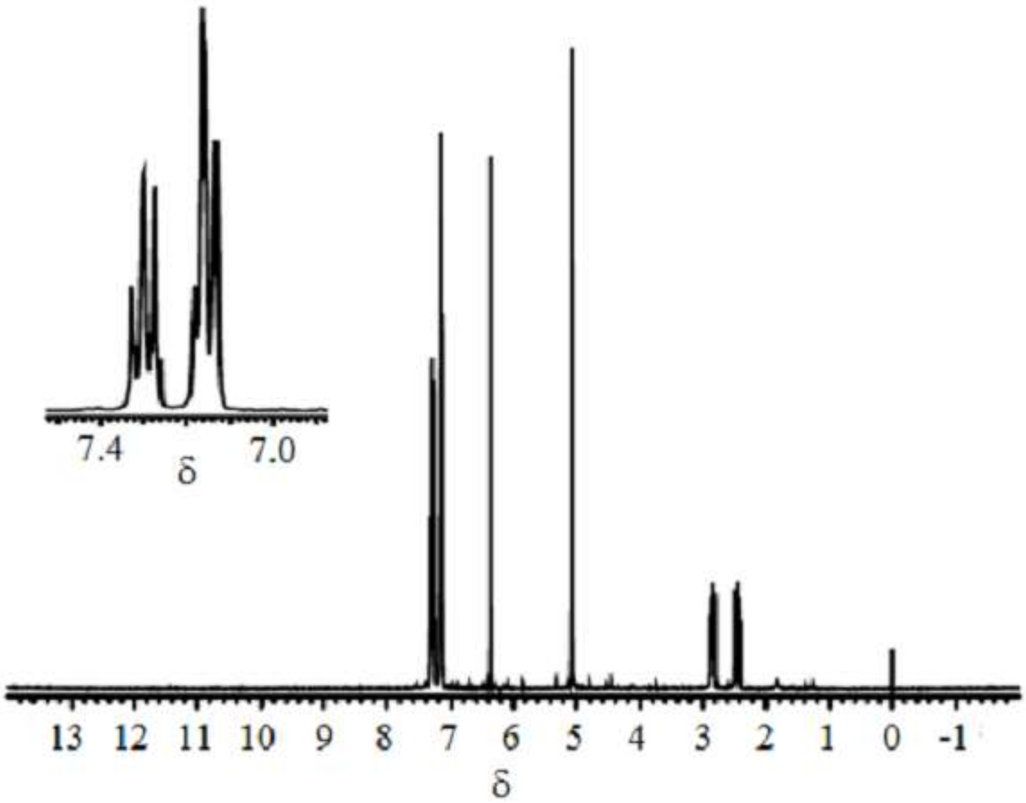

Figure S35. The ^{1}H NMR Spectrum for Di[(3-diphenylphosphonatopropanoyl)methyl]furan (DPMF)

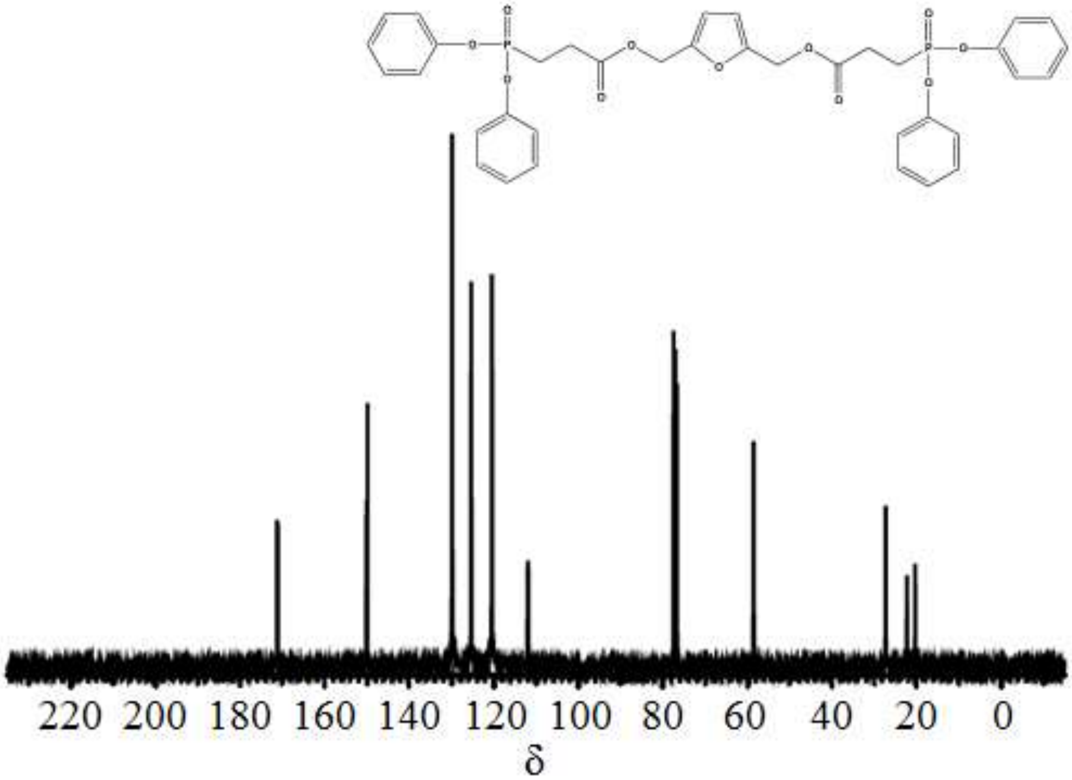

Figure S36. The ^{13}C NMR Spectrum for Di[(3-diphenylphosphonatopropanoyl)methyl]furan (DPMF)

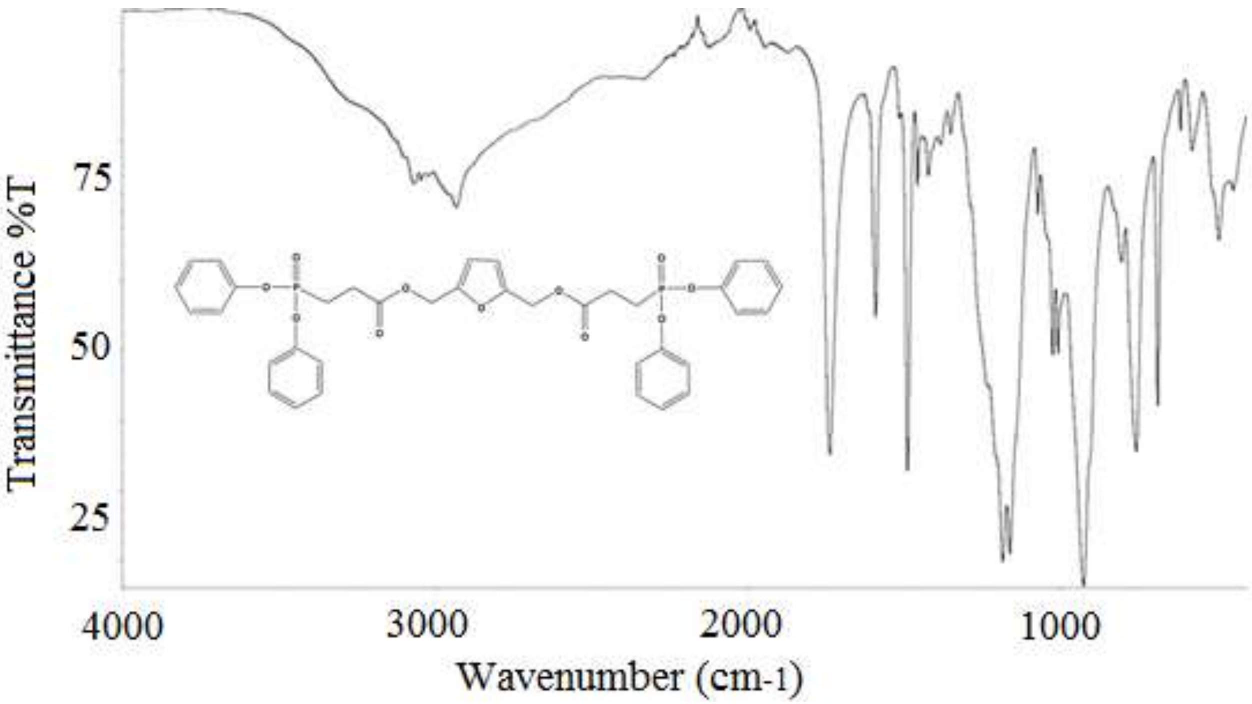

Figure S37. The Infrared Spectrum for Di[(3-diphenylphosphonatopropanoyl)methyl]furan (DPMF)

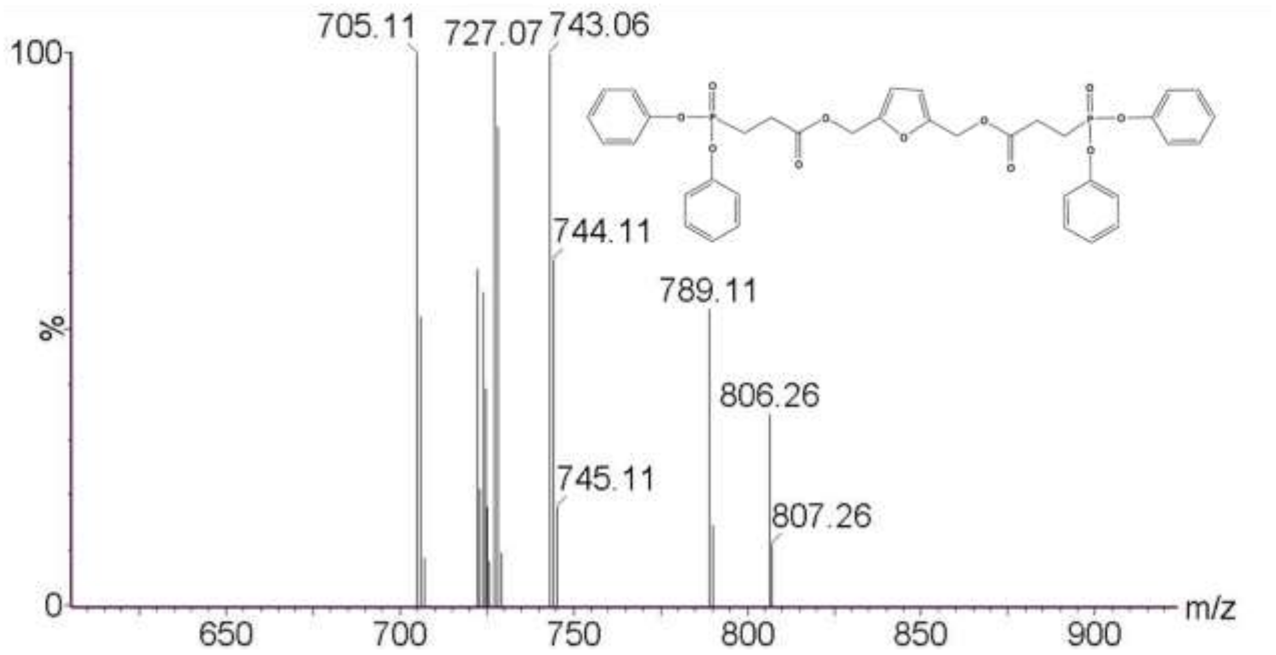

Figure S38. The ESI Mass Spectrum for Di[(3-diphenylphosphonatopropanoyl)methyl]furan (DPMF)
